# Gut microbiota-liver-kidney axis in diabetic kidney disease: mechanistic insights into amino acid metabolism and nutritional intervention strategies targeting natural bioactive compounds

**DOI:** 10.3389/fnut.2026.1778473

**Published:** 2026-04-28

**Authors:** Li-ya Sun, Gui-yan Sun, Yang Nan, Hao-ran Wang, Yue Sun, Yan Shi, Yu-feng Yang, Qing-feng Wang

**Affiliations:** 1 Liaoning University of Traditional Chinese Medicine, Shenyang, China; 2 Liaoning Academy of Traditional Chinese Medicine (The Second Affiliated Hospital of Liaoning University of Traditional Chinese Medicine), Shenyang, China

**Keywords:** amino acid metabolism, diabetic kidney disease, functional foods, gut microbiota, gut-liver-kidney axis, medicine food homology, natural bioactive compounds, uremic toxins

## Abstract

Diabetic kidney disease (DKD) is a leading cause of end-stage renal disease globally. Emerging research highlights the gut microbiota-gut-liver-kidney axis as a critical metabolic nexus linking dietary intake to DKD pathogenesis and progression. The gut microbiota, acting as a vast metabolic organ, transforms dietary components into key metabolites. Beneficial fermentation of fiber produces short-chain fatty acids (SCFAs) like butyrate, which exert anti-inflammatory and renal protective effects. Conversely, microbial metabolism of aromatic amino acids generates protein-bound uremic toxins, such as indoxyl sulfate and p-cresyl sulfate, which promote oxidative stress, inflammation, and fibrosis upon renal accumulation. DKD is characterized by intestinal barrier dysfunction (“leaky gut”) and metabolic endotoxemia, creating a vicious cycle that sustains systemic inflammation and kidney injury. Nutritional interventions targeting this axis show therapeutic promise. Dietary patterns (e.g., Mediterranean diet, increased plant protein) and specific prebiotics can modulate microbial composition, enhance SCFA production, and reduce uremic toxins. Natural bioactive compounds (e.g., berberine, quercetin, astragalus polysaccharides) and “medicine food homology” substances demonstrate multi-target renoprotective effects by restoring microbiota balance, improving intestinal barrier integrity, and mitigating metabolic dysregulation. Future management strategies will leverage precision nutrition, utilizing multi-omics and artificial intelligence to design personalized dietary interventions based on individual microbiota profiles, offering a novel paradigm for comprehensive DKD care alongside conventional therapies.

## 1 Introduction

Diabetic kidney disease (DKD) is one of the most common and severe microvascular complications of diabetes mellitus, representing the leading cause of end-stage renal disease (ESRD) worldwide (1). According to the latest data from the International Diabetes Federation (IDF), the global prevalence of diabetes increased dramatically from 151 million in 1990 to 537 million in 2021, with projections suggesting a further rise to 783 million by 2045 (2). As diabetes prevalence continues to escalate, the disease burden of DKD is becoming increasingly substantial. Epidemiological studies indicate that approximately 20%−40% of diabetic patients develop DKD during the course of their disease, with a considerable proportion ultimately requiring renal replacement therapy (3). The Global Burden of Disease Study 2021 demonstrated that the burden of diabetes-attributable chronic kidney disease (CKD) showed a significant upward trend from 1990 to 2021, and this trajectory is projected to continue through 2050 (4).

The pathogenesis of DKD is extremely complex, involving dysregulation of multiple metabolic pathways. Chronic hyperglycemia induces excessive accumulation of advanced glycation end products (AGEs), enhanced oxidative stress, activation of inflammatory responses, and aberrant activation of the renin-angiotensin system (RAS). These factors act synergistically to cause glomerular filtration barrier damage and tubulointerstitial fibrosis, ultimately leading to irreversible decline in renal function (5). Notably, nutritional metabolic disorders occupy a central position in the development and progression of DKD. Research has demonstrated that DKD patients commonly exhibit lipid metabolism abnormalities, amino acid metabolic imbalances, and uremic toxin accumulation, which not only accelerate renal injury progression but are also closely associated with increased cardiovascular disease risk (6).

The association between dietary factors and DKD has been supported by substantial epidemiological evidence. High-protein diets can increase renal metabolic burden and promote the generation of harmful metabolites derived from intestinal protein fermentation, while Western dietary patterns characterized by high fat and sugar intake are closely associated with insulin resistance, chronic low-grade inflammation, and gut microbiota dysbiosis (7). Conversely, healthy dietary patterns rich in fiber and phytochemicals have been shown to delay DKD progression. A case-control study found that higher phytochemical index (PI) intake was significantly associated with reduced DKD risk (OR = 0.15, 95% CI: 0.06–0.36) (8). Furthermore, cross-sectional analyses based on the National Health and Nutrition Examination Survey (NHANES) confirmed an inverse association between dietary fiber intake and DKD prevalence (9). These findings provide important evidence for exploring DKD prevention and treatment strategies centered on nutritional intervention.

The human intestinal tract is colonized by trillions of microorganisms that participate in various physiological processes including food digestion, synthesis of essential vitamins and amino acids, pathogen antagonism, and toxin clearance (10). In recent years, the relationship between gut microbiota and host metabolic health has attracted increasing attention. Extensive research has demonstrated that alterations in gut microbiota composition and function are closely associated with the development and progression of type 2 diabetes and its complications (11). DKD patients exhibit significant structural dysbiosis of gut microbiota, characterized by decreased abundance of beneficial bacteria producing short-chain fatty acids (SCFAs) and increased abundance of harmful bacteria generating uremic toxin precursors (12).

A close triangular interaction exists among diet, gut microbiota, and host metabolism. Dietary composition is a key determinant of gut microbiota composition, with different nutrients selectively promoting or inhibiting the growth of specific microbial populations. Reciprocally, gut microbiota biotransform dietary components to produce various physiologically active metabolites, such as SCFAs, secondary bile acids, and indole derivatives, which enter the liver via the portal venous system and participate in host glucose and lipid metabolism as well as immune regulation (13). Among gut microbiota-derived metabolites, bacterial tryptophan metabolites such as indole-3-propionic acid (IPA) can improve intestinal barrier function and insulin sensitivity through activation of the aryl hydrocarbon receptor (AhR) (14); meanwhile, increased microbial biosynthesis of branched-chain amino acids (BCAAs) is closely associated with insulin resistance (15).

The concept of the “gut-liver-kidney” nutritional metabolic axis has gradually emerged and received widespread attention in recent years. Intestinal barrier dysfunction and dysbiosis can lead to translocation of bacterial lipopolysaccharide (LPS) and gut-derived toxins into the systemic circulation, triggering systemic low-grade inflammation (16). After reaching the liver via the portal vein, some of these toxins are metabolized and detoxified by the liver, while others are excreted through the kidneys. During DKD progression, gut microbiota-derived uremic toxins such as indoxyl sulfate (IS) and p-cresyl sulfate (pCS) accumulate in the body due to impaired renal clearance, further exacerbating oxidative stress and inflammatory injury in the kidneys, thus forming a vicious cycle (17). Studies have confirmed that gut microbiota dysbiosis participates in DKD development and progression through multiple mechanisms including metabolic endotoxemia, aberrant SCFA metabolism, and oxidative stress (18). Therefore, therapeutic strategies targeting the gut microbiota-enterohepatic axis hold promise for opening new avenues in DKD prevention and treatment.

Traditional nutritional management of DKD has primarily focused on single-nutrient interventions such as protein restriction, glycemic control, and blood pressure management. However, with deepening understanding of disease pathogenesis, researchers have increasingly recognized the importance of transitioning from single-nutrient approaches to holistic dietary pattern modifications. Dietary patterns rich in plant-based foods, fiber, and phytochemicals not only improve glycemic control but also exert renoprotective effects through multiple mechanisms including gut microbiota modulation, reduction of uremic toxin generation, and enhancement of antioxidant defense (19). Animal experiments have demonstrated that dietary fiber can delay DKD progression by promoting the proliferation of SCFA-producing bacteria, activating G protein-coupled receptors GPR43 and GPR109A signaling pathways, and inhibiting high glucose-induced oxidative stress and NF-κB inflammatory signaling (20).

Research on natural bioactive compounds and functional foods has become a hot topic in the field of DKD nutritional intervention. Plant-derived bioactive substances, including polyphenols (flavonoids, phenolic acids, anthocyanins), polysaccharides (inulin, fructooligosaccharides, plant polysaccharides), saponins (ginsenosides, astragaloside IV), and alkaloids (berberine), have been shown to possess multiple biological activities such as gut microbiota modulation, intestinal barrier improvement, reduction of inflammatory factor release, and antioxidant effects (21). A systematic bibliometric analysis revealed that research on natural products for DKD treatment has grown exponentially in recent years, with research hotspots primarily focusing on oxidative stress, inflammatory responses, gut microbiota regulation, and podocyte protection (22). Traditional Chinese medicine has accumulated rich experience in DKD treatment, with traditional formulas and natural active monomers demonstrated to improve clinical symptoms in DKD patients through multi-target, multi-pathway holistic regulation (23).

Probiotics and prebiotics, as nutritional intervention approaches for gut microbiota modulation, have shown promising application prospects in DKD management. Results from randomized controlled trials and meta-analyses indicate that probiotic supplementation can significantly improve glucose and lipid metabolism, alleviate renal function impairment, and reduce inflammation and oxidative stress levels in DKD patients (24). Fecal microbiota transplantation (FMT), as an emerging microbiota reconstruction strategy, has also demonstrated potential therapeutic value in DKD animal models (25).

This review aims to systematically elucidate the central hub role of the gut microbiota-enterohepatic axis in dietary nutrient metabolism, with emphasis on exploring the association mechanisms between gut microbiota-mediated amino acid metabolism and DKD, and reviewing research progress on natural bioactive compounds targeting the gut microbiota-enterohepatic axis for DKD intervention. By integrating basic research and clinical evidence, this article seeks to provide new perspectives for DKD nutritional intervention strategies and theoretical foundations for the development and application of functional foods and medicinal-edible homologous substances.

## 2 Literature search strategy

This review employed a systematic literature search methodology, searching databases including PubMed, Web of Science, Embase, and China National Knowledge Infrastructure (CNKI), with a search period from database inception to November 2024. The search strategy combined MeSH terms and free-text words. English search terms included: “diabetic kidney disease” OR “diabetic nephropathy” OR “DKD” OR “DN,” combined with “gut microbiota” OR “gut microbiome” OR “intestinal flora” OR “intestinal microbiota,” “dietary” OR “nutrition” OR “diet” OR “nutritional intervention,” “phytochemical” OR “natural product” OR “bioactive compound” OR “functional food,” “amino acid metabolism” OR “metabolite” OR “metabolomics,” “gut-liver axis” OR “gut-kidney axis” OR “enterohepatic axis,” “short-chain fatty acids” OR “SCFAs,” and “probiotics” OR “prebiotics” OR “synbiotics.”

Inclusion criteria were: (1) studies addressing the associations among DKD, gut microbiota, nutritional intervention, and natural bioactive compounds; (2) publication types including original research articles, systematic reviews, meta-analyses, and clinical trials; (3) language restricted to Chinese and English; and (4) study subjects including humans, animal models, or *in vitro* cell experiments. Exclusion criteria were: (1) conference abstracts, theses, and case reports; (2) duplicate publications; (3) articles with inaccessible full text; and (4) articles with low relevance to the topic. The literature screening process was independently completed by two researchers, with disagreements resolved through discussion or consultation with a third researcher. Additionally, reference lists of included articles were manually searched to ensure comprehensiveness.

## 3 The gut microbiota-gut-liver axis: a central hub for dietary nutrient metabolism

### 3.1 Interactions between the gut microbiota and dietary nutrients

#### 3.1.1 Dietary patterns shape gut microbiota composition

The human gastrointestinal tract harbors approximately 100 trillion microbial cells, comprising over 1,000 bacterial species, with a collective genome encoding approximately 150 times more genes than the human genome (26). This complex microbial community constitutes the body's largest “metabolic organ,” with its composition and function profoundly influenced by dietary structure (27). Studies have demonstrated that dietary patterns represent the predominant environmental factor shaping gut microbiota composition, with an influence exceeding that of host genetics (28).

Long-term dietary habits significantly alter gut microbiota structural characteristics. High-fiber, plant-based diets promote the enrichment of short-chain fatty acid (SCFA)-producing bacteria, particularly genera within the Firmicutes phylum including Faecalibacterium, Roseburia, and Eubacterium (29). In contrast, the Western dietary pattern (high fat, high sugar, low fiber) leads to decreased intestinal microbial diversity, elevated Firmicutes/Bacteroidetes ratio, and impaired intestinal barrier function (30). Jardon et al. (31) demonstrated through precision nutrition research that adjustments in dietary macronutrient proportions can induce significant changes in gut microbiota composition within days, providing a theoretical foundation for improving metabolic health through dietary intervention.

The Mediterranean diet, recognized as a healthy dietary pattern, has been demonstrated to improve gut microbiota richness, composition, and function (32). A landmark intervention trial in diabetic patients found that a high mixed-fiber diet selectively promoted the proliferation of SCFA-producing bacterial strains, particularly enriching bacteria carrying carbohydrate-active enzyme-encoding genes, thereby significantly improving glycated hemoglobin levels and postprandial glucose responses (33). These findings underscore the central role of diet-microbiota interactions in metabolic regulation.

#### 3.1.2 Biotransformation of dietary components by the gut microbiota

The gut microbiota possesses extensive metabolic capabilities, transforming dietary components into numerous bioactive metabolites. These metabolites can be categorized into three classes: (1) metabolites produced from bacterial degradation of nutrients, such as SCFAs; (2) host-derived metabolites modified by the microbiota, such as secondary bile acids; and (3) metabolites synthesized *de novo* by the microbiota, such as lipopolysaccharide (LPS) (34). This metabolic network positions the gut microbiota as a critical intermediary between dietary nutrients and host metabolism.

The metabolic capacity of the gut microbiota compensates for deficiencies in the human enzyme system. Humans lack enzymes capable of degrading complex carbohydrates and thus rely on gut bacteria to hydrolyze and ferment dietary fiber and resistant starch, producing monosaccharides and SCFAs (35). It is estimated that microbial fermentation produces 400–600 mmol of SCFAs daily, providing approximately 10% of the body's energy requirements (36). Additionally, the gut microbiota participates in the synthesis of essential vitamins, including vitamin K, biotin, folate, and various B vitamins (37).

Microbial metabolites influence host physiological functions through multiple mechanisms. Studies have demonstrated that microbiota-derived SCFAs serve as natural ligands for free fatty acid receptors 2/3 (FFAR2/3), activating signaling pathways in enteroendocrine cells, immune cells, and other cell types (38). Simultaneously, the microbiota deconjugates primary bile acids via bile salt hydrolase (BSH) and converts them to secondary bile acids through 7α-dehydroxylase activity, modifications that significantly affect bile acid signaling functions (39).

#### 3.1.3 Microbial metabolism of dietary fiber, protein, and fat

Dietary fiber metabolism: non-digestible carbohydrates represent the primary fermentation substrates for gut bacteria. Among anaerobic fermentation products, acetate, propionate, and butyrate are the predominant SCFAs, with a molar ratio of approximately 60:20:20 (40). Different types of dietary fiber exhibit distinct fermentation characteristics. Studies have shown that inulin-type fructans (ITF) and resistant starch (RS) increase butyrate production potential by enriching bacteria carrying the butyryl-CoA:acetate-CoA transferase (but) gene (41). β-glucan intake promotes the proliferation of Bacteroides and Bifidobacterium while increasing the abundance of butyrate-producing Clostridium species (42).

Butyrate, serving as the primary energy source for colonocytes, possesses multiple functions including maintaining intestinal barrier integrity, modulating immune responses, and suppressing inflammation (43). Mann et al. (38) noted that butyrate directly affects the differentiation of intestinal epithelial cells, phagocytes, B cells, regulatory T cells, and effector T cells, representing a key signaling molecule linking diet, microbiota, and the immune system.

Protein metabolism: in modern diets, the quantity of protein entering the colon is approximately equivalent to that of carbohydrates (44). Protein not absorbed in the small intestine undergoes microbial fermentation in the colon, producing branched-chain fatty acids (BCFAs), ammonia, hydrogen sulfide, and phenolic compounds. Jackson et al. (45) found that when dietary protein is co-fermented with fiber, butyrate production increases rather than decreases, suggesting that undigested protein may promote microbial butyrate synthesis in fiber-rich substrates.

Tryptophan, an essential amino acid, is metabolized by gut bacteria to produce indole and indole derivatives that serve as aryl hydrocarbon receptor (AhR) ligands, participating in the regulation of intestinal barrier function and immune homeostasis (14). Additionally, microbial fermentation products such as succinate participate in energy homeostasis regulation through the gut-brain axis (46).

Fat metabolism: dietary fat primarily comprises triglycerides, with minor amounts of phospholipids, sphingolipids, and cholesterol. Microbial lipases hydrolyze triglycerides and phospholipids to polar head groups and free fatty acids (47). High-fat diets significantly alter gut microbiota composition, leading to elevated Firmicutes/Bacteroidetes ratios, increased Enterobacteriaceae abundance, and enhanced LPS release from Gram-negative bacteria, triggering low-grade chronic inflammation (48).

Recent studies have confirmed that the gut microbiota directly participates in cholesterol metabolism. Li et al. (49) discovered that Oscillibacter species can metabolize cholesterol through dehydrogenation and glycosylation reactions, while Bacteroides thetaiotaomicron correlates positively with plasma cholesterol and cholesterol sulfate levels. These findings reveal direct microbial roles in lipid metabolism.

#### 3.1.4 Human validation studies and translational considerations for key bacterial genera

##### 3.1.4.1 Genera identified by mendelian randomization studies

Mendelian randomization (MR) studies have identified multiple gut bacterial genera associated with diabetic kidney disease (DKD) risk. Akkermansia genus was associated with increased DKD risk (OR = 1.45, 95% CI: 1.18–1.79), while Dialister genus and Proteobacteria phylum showed protective effects. These findings suggest specific genera may influence DKD occurrence through metabolic regulation.

However, causal inferences from MR studies require validation through longitudinal cohort studies. Cross-sectional studies showed that Agathobacter and Prevotella_9 abundance correlated positively with eGFR and negatively with microalbuminuria(50). Ruminococcus_gnavus abundance correlated positively with 24-h urinary protein and serum creatinine (50). These genera can serve as ROC curve discriminators, with Agathobacter and Prevotella_9 achieving AUCs of 89.96 and 83.42%, respectively.

##### 3.1.4.2 Translational difficulties between animal models and human studies

Approximately 65% of studies cited in this review are based on animal models (primarily db/db mice and STZ-induced diabetic rats), 25% are human cross-sectional studies, and only 10% are human prospective cohort studies. Significant differences exist between animals and humans in gut microbiota: (1) Microbiota composition differences: rodent intestines have similar dominant phyla to humans, but genus-level composition differs significantly. *Akkermansia muciniphila* comprises 3%−5% of mouse gut microbiota but only 1%−3% in humans (51); (2) Metabolic pathway differences: certain microbial metabolites observed in animal models (such as specific indole derivatives) may differ in production levels and metabolic pathways in humans (52); and (3) Disease model limitations: STZ models primarily simulate β-cell destruction in type 1 diabetes, whereas most DKD patients have type 2 diabetes with fundamentally different pathological mechanisms (53).

##### 3.1.4.3 Strategies to bridge translational difficulties

To improve clinical translatability, the following strategies are recommended: (1) Multi-species validation: mechanisms discovered in mice should be validated in rats, rabbits, or non-human primates; (2) Human microbiota transplantation models: germ-free mice transplanted with fecal microbiota from human DKD patients (HFA model) can more realistically simulate human microbiota-host interactions (33); (3) Prospective cohort studies: large-scale, multicenter prospective cohorts are needed to longitudinally track microbiota changes and their relationship with DKD progression; (4) Standardized detection: establish standardized procedures for gut microbiota detection and analysis to improve study comparability; and (5) Population-specific validation: considering microbiota composition differences across populations (East Asian vs. Western), population-specific studies should be conducted.

### 3.2 Nutritional metabolic functions of the gut-liver axis

#### 3.2.1 The portal venous system: the “first station” for nutrients

The gut-liver axis establishes a direct anatomical connection between the intestine and liver through the portal venous system. Nutrients absorbed in the intestine and microbial metabolites are collected via the superior and inferior mesenteric veins into the portal vein, entering the liver directly for “first-pass metabolism.” This unique circulatory pathway positions the liver as the “first station” for processing gut-derived substances (54).

The portal venous system carries multiple signaling molecules from the intestine. Among SCFAs, acetate and propionate primarily enter the liver via the portal vein, serving as precursors for fatty acid synthesis and gluconeogenesis, respectively; butyrate is predominantly utilized by colonocytes, with only small amounts entering systemic circulation (55). Hepatic processing of these metabolites directly influences whole-body energy metabolism and glucose homeostasis. Studies have demonstrated that propionate is efficiently utilized by the liver as a gluconeogenic substrate, while acetate participates in hepatic *de novo* lipogenesis (DNL) (56).

When intestinal barrier function is compromised, bacterial components such as LPS can enter the liver via the portal vein, triggering Toll-like receptor 4 (TLR4)-mediated inflammatory responses (57). This pathological process, termed “metabolic endotoxemia,” represents a key mechanism linking gut microbiota dysbiosis with metabolic diseases. High-fat diet feeding in mice for 4 weeks can elevate plasma LPS levels 2–3 fold, accompanied by Proteobacteria expansion (58).

#### 3.2.2 Hepatic processing and distribution of gut-derived metabolites

The liver, as the body's largest metabolic organ, possesses robust capabilities for substrate extraction and biotransformation. It processes, modifies, and distributes gut-derived metabolites to maintain systemic metabolic homeostasis. The hepatocyte cytochrome P450 enzyme system represents the principal metabolic machinery, participating in oxidation, reduction, hydrolysis, and conjugation reactions of various exogenous and endogenous substances (59).

SCFAs undergo distinct metabolic fates upon entering the liver. Propionate enters the gluconeogenic pathway via propionyl-CoA carboxylase, providing substrate for fasting blood glucose maintenance; acetate is converted to acetyl-CoA, participating in fatty acid and cholesterol synthesis (60). Münte et al. (61) reported that SCFAs promote fatty acid oxidation through AMP-activated protein kinase (AMPK) activation while inhibiting lipoprotein lipase activity, thereby modulating fatty acid uptake and storage in adipose tissue.

The liver is also the exclusive site of bile acid synthesis. Cholesterol 7α-hydroxylase (CYP7A1) is the rate-limiting enzyme in the classical bile acid synthesis pathway, metabolizing approximately 90% of cholesterol daily (62). Primary bile acids (cholic acid and chenodeoxycholic acid) synthesized by the liver are conjugated with glycine or taurine before secretion into bile, stored in the gallbladder, and released into the intestine postprandially to facilitate absorption of lipid-soluble nutrients.

#### 3.2.3 Bile acid enterohepatic circulation and regulation of nutrient absorption

The enterohepatic circulation of bile acids represents a core pathway connecting intestinal and hepatic metabolism. Bile acids absorbed in the intestine return to the liver via the portal vein, forming an efficient recycling system that cycles 6–8 times daily, with only approximately 5% excreted in feces (63). This circulation maintains bile acid pool stability while controlling bile acid synthesis rates through feedback regulation.

As physiological detergents, bile acids are essential for the absorption of dietary fat, steroids, and fat-soluble vitamins. They form mixed micelles with lipids in the intestinal lumen, facilitating the solubilization and transmembrane transport of fatty acids, monoglycerides, and cholesterol (64). Bile acids also possess direct antimicrobial activity; their detergent-like properties can disrupt bacterial cell membranes, causing cell lysis and thereby modulating gut microbiota composition (65).

**Figure 1:**
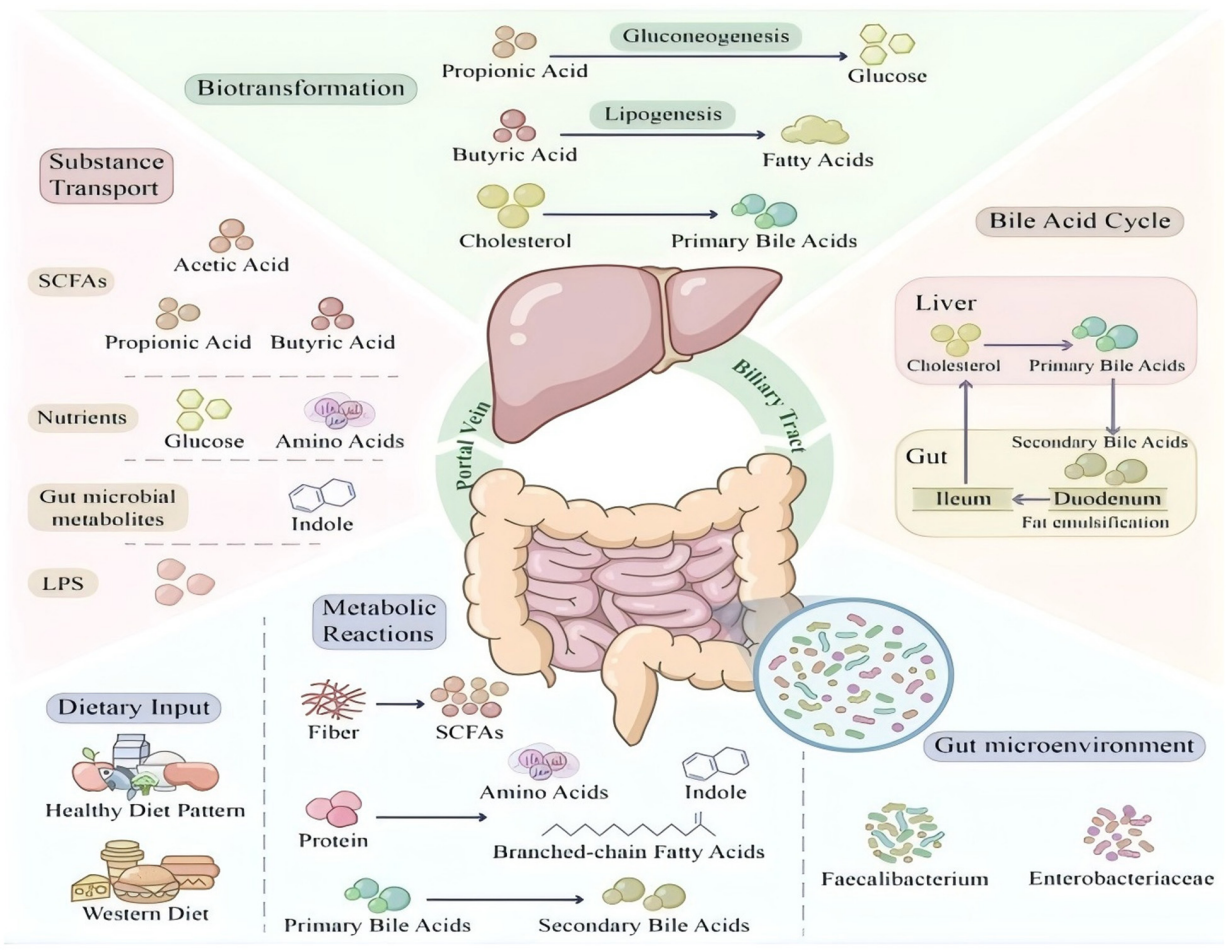
The gut-liver metabolic axis illustrating the bidirectional communication between intestinal microbiota and hepatic metabolism. This schematic diagram depicts the intricate metabolic crosstalk between the gut and liver via the portal vein and biliary system. **(Left panel)**: the intestinal compartment shows dietary inputs (fiber-rich diet vs. Western diet) and their differential effects on gut microbiota composition. Beneficial bacteria (*Faecalibacterium, Roseburia*) ferment dietary fiber to produce short-chain fatty acids (SCFAs: acetate, propionate, butyrate), while pathogenic bacteria (*Enterobacteriaceae*) predominate under Western diet conditions. Primary bile acids undergo 7α-dehydroxylation by gut microbiota to form secondary bile acids. **(Right panel)**: the liver receives portal blood containing SCFAs, microbial metabolites (indoles), nutrients, and potentially lipopolysaccharide (LPS) under dysbiotic conditions. Hepatocytes process these substrates: propionate undergoes gluconeogenesis, acetate serves as substrate for lipogenesis, and cholesterol is converted to primary bile acids. SCFAs, short-chain fatty acids; LPS, lipopolysaccharide.

Microbial modification of bile acids is an important component of gut-liver axis function. In the terminal ileum and colon, bacterial BSH first deconjugates conjugated bile acids, releasing taurine or glycine; subsequently, bacterial 7α-dehydroxylase converts primary bile acids to secondary bile acids (deoxycholic acid and lithocholic acid) (66). These microbial modifications significantly alter the physicochemical properties and signaling capacity of bile acids, thereby influencing host metabolism. The bidirectional communication between the gut and liver through the portal vein and biliary system establishes a metabolic axis that integrates dietary nutrient processing with systemic metabolic homeostasis (Figure 1).

### 3.3 Signaling regulatory networks of the gut microbiota-gut-liver axis

#### 3.3.1 Bile acid receptor (FXR, TGR5) signaling pathways

Bile acids function not only as digestive molecules but also as important metabolic regulatory signaling molecules. They primarily exert metabolic integration functions through activation of the nuclear receptor farnesoid X receptor (FXR) and the membrane G protein-coupled bile acid receptor 5 (TGR5) (67).

FXR is widely expressed in the liver and intestine and serves as a feedback sensor for bile acid synthesis. Upon bile acid activation, hepatic FXR induces expression of small heterodimer partner (SHP), which suppresses CYP7A1 and CYP8B1 gene transcription, thereby reducing bile acid synthesis (68). Intestinal FXR activation induces expression and secretion of fibroblast growth factor 19 (FGF19; FGF15 in mice), which enters the liver via the portal vein and activates the FGF receptor 4 (FGFR4)/β-Klotho complex, further suppressing CYP7A1 transcription through the ERK1/2 signaling pathway (69).

FXR also participates in regulating glucose and lipid metabolism. Wang et al. (70) demonstrated that dual FXR and TGR5 agonists can reverse Western diet-induced hepatic steatosis, inflammation, and fibrosis in mice. FXR activation suppresses expression of hepatic gluconeogenic and lipogenic genes, improving hyperglycemia and hyperlipidemia (71).

TGR5 is a membrane G protein-coupled receptor widely expressed in the intestine, gallbladder, hepatic sinusoidal endothelial cells, and Kupffer cells. Secondary bile acids (lithocholic acid and deoxycholic acid) are its most potent agonists (72). Upon TGR5 activation, adenylyl cyclase (AC) converts ATP to cAMP, which subsequently activates protein kinase A (PKA) and cAMP response element-binding protein (CREB), participating in multiple cAMP signaling pathways (73).

In the intestine, TGR5 activates enteroendocrine L cells, stimulating glucagon-like peptide-1 (GLP-1) secretion (74). GLP-1 enters circulation and promotes insulin secretion from pancreatic β-cells, improving glucose tolerance and insulin sensitivity. In brown adipose tissue and skeletal muscle, TGR5 induces type 2 deiodinase (DIO2) expression, converting thyroid hormone T4 to T3 and stimulating mitochondrial energy metabolism (75). Additionally, TGR5 exerts anti-inflammatory effects by inhibiting NF-κB-mediated pro-inflammatory cytokine production and modulating NLRP3 inflammasome activation (76).

#### 3.3.2 Metabolic regulatory functions of short-chain fatty acids (SCFAs)

As the primary fermentation products of dietary fiber, SCFAs serve as core mediators of communication between gut microbiota and host metabolism. They regulate local intestinal function and systemic metabolism through multiple mechanisms (77).

Energy metabolism regulation: SCFAs provide 60%−70% of the energy requirements for colonocytes, with butyrate being the preferred substrate (60). SCFAs not utilized by the intestine enter the liver via the portal vein, participating in gluconeogenesis and lipogenesis. Propionate serves as a gluconeogenic precursor, promoting hepatic glucose production; acetate, as a precursor to acetyl-CoA, participates in *de novo* fatty acid and cholesterol synthesis (78). Studies have demonstrated that SCFAs promote fatty acid oxidation and energy expenditure through AMPK activation while inhibiting lipoprotein lipase activity, reducing fatty acid release (79).

Receptor-mediated signal transduction: SCFAs are natural ligands for FFAR2 and FFAR3, receptors widely expressed in enteroendocrine and immune cells (80). SCFA binding to FFAR2/3 stimulates enteroendocrine cells to release peptide YY (PYY) and GLP-1, hormones that regulate appetite and energy intake through the gut-brain axis (81). Hays et al. (82) noted that SCFAs have profound effects on appetite, energy balance, and both innate and adaptive immunity, with actions extending far beyond the intestine itself.

Immune regulatory functions: SCFAs exert epigenetic regulatory effects through histone deacetylase (HDAC) inhibition, influencing immune cell differentiation and function (83). Butyrate promotes the differentiation of regulatory T cells (Tregs), enhancing their immunosuppressive function and maintaining intestinal immune tolerance (84). Additionally, SCFAs regulate type 3 innate lymphoid cells (ILC3) through GPR43 activation, promoting antimicrobial peptide and IL-22 release to enhance intestinal mucosal barrier function (85).

#### 3.3.3 Intestinal barrier integrity and metabolic endotoxemia

The intestinal barrier represents a selectively permeable barrier between the internal environment and intestinal luminal contents, comprising mechanical barriers (epithelial cells and tight junctions), chemical barriers (mucus layer and antimicrobial peptides), immune barriers, and biological barriers (commensal microbiota) (86). Barrier integrity is crucial for preventing the invasion of harmful substances and pathogens.

Intestinal barrier dysfunction, termed “leaky gut,” is characterized by increased translocation of bacterial metabolites and endotoxins (particularly LPS) into systemic circulation (87). Di Vincenzo et al. (88) noted that LPS release due to barrier disruption is closely associated with the development and progression of multiple metabolic diseases, including obesity, type 2 diabetes, non-alcoholic fatty liver disease, and cardiovascular disease.

High-fat diet is a major factor inducing intestinal barrier dysfunction. Animal experiments have demonstrated that high-fat diet downregulates tight junction protein expression (such as occludin and ZO-1), reduces mucus layer thickness, and increases intestinal permeability (89). Zhang et al. (90) confirmed that patients with obesity, NAFLD, and diabetes all exhibit significantly increased intestinal permeability, with mechanisms involving increased intestinal pathogenic bacteria and inhibitory effects of hyperglycemia and hyperlipidemia on tight junction protein expression.

Metabolic endotoxemia refers to a state of chronically elevated circulating LPS levels caused by diet (particularly high-fat diet) (91). The classic study by Cani et al. (92) first proposed this concept, finding that high-fat diet mice exhibited significantly elevated plasma LPS levels accompanied by increased Gram-negative bacteria proportions and enhanced intestinal permeability. LPS activates NF-κB signaling through the TLR4/CD14-MyD88-dependent pathway, inducing excessive production of pro-inflammatory cytokines such as TNF-α and IL-6, triggering systemic low-grade chronic inflammation (93).

This inflammatory state further damages intestinal barrier function, forming a vicious cycle. Dmytriv et al. (94) noted that excessive intestinal permeability promotes inflammatory responses, while inflammation-generated pro-inflammatory cytokines and reactive oxygen species (ROS) exacerbate barrier dysfunction. Fortunately, dietary intervention can break this vicious cycle. Diets rich in plant polyphenols or fermented dairy products can increase tight junction protein expression, promote beneficial bacterial growth, and improve intestinal barrier function (95).

**Figure 2:**
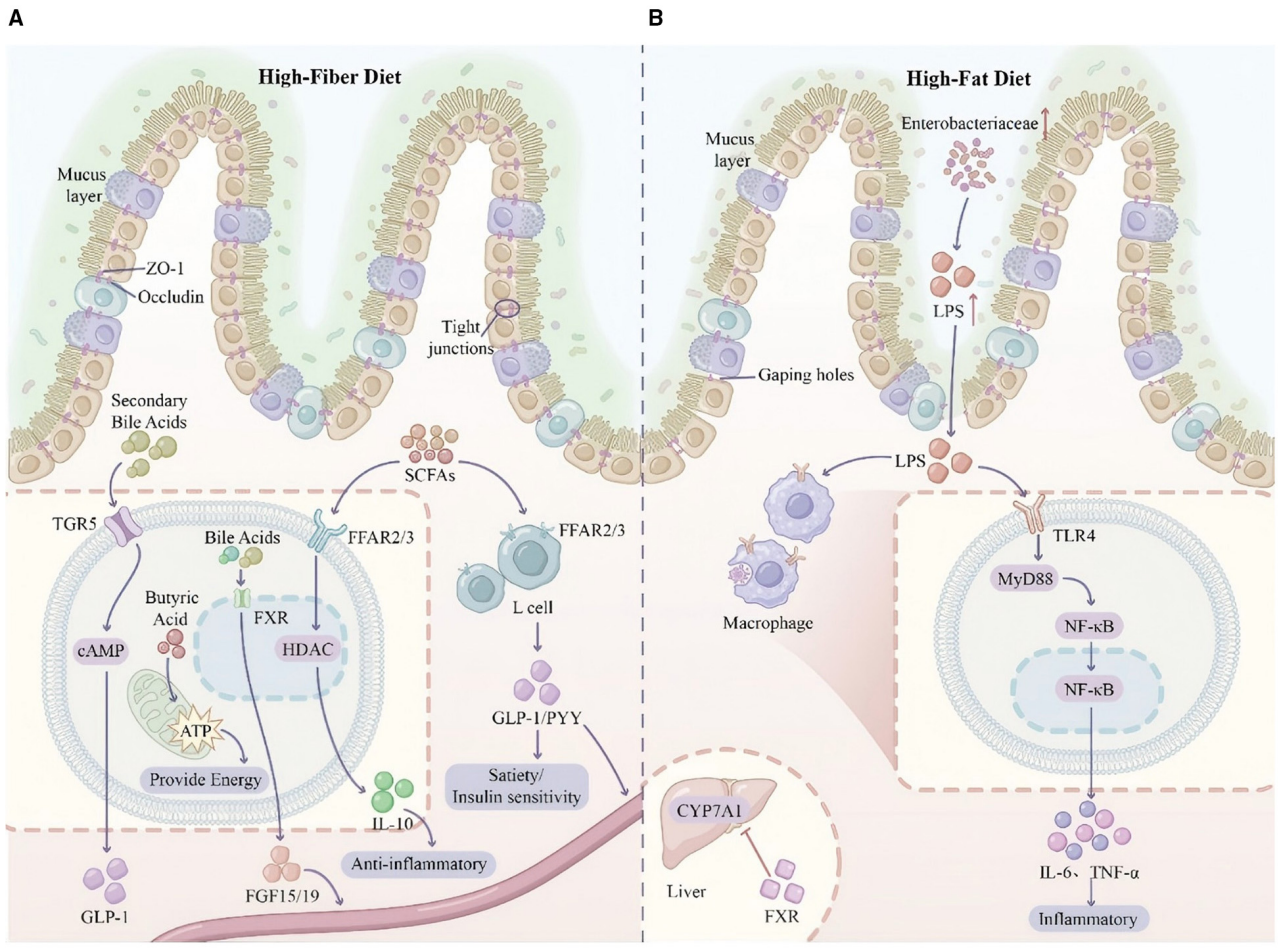
Molecular signaling networks and intestinal barrier function under homeostatic and dysbiotic conditions. This split-screen diagram contrasts the molecular mechanisms governing intestinal barrier integrity in health vs. disease states. **Left panel (homeostasis)**: under high-fiber dietary conditions, SCFAs activate free fatty acid receptors 2/3 (FFAR2/3) on enteroendocrine L-cells, stimulating glucagon-like peptide-1 (GLP-1) and peptide YY (PYY) secretion, thereby enhancing satiety and insulin sensitivity. In regulatory T cells (Tregs), SCFAs inhibit histone deacetylase (HDAC), promoting interleukin-10 (IL-10) production and anti-inflammatory responses. Secondary bile acids activate Takeda G protein-coupled receptor 5 (TGR5), elevating cyclic AMP and augmenting GLP-1 release. Farnesoid X receptor (FXR) activation by bile acids induces fibroblast growth factor 15/19 (FGF15/19) secretion for hepatic feedback regulation. The intestinal barrier maintains integrity through robust tight junction proteins (ZO-1, occludin) and a thick mucus layer supported by *Akkermansia muciniphila*. Butyrate provides colonocyte energy via mitochondrial β-oxidation. **Right panel (dysbiosis/inflammation)**: high-fat diet-induced alterations in bile acid composition promote Gram-negative bacterial overgrowth and LPS accumulation. Barrier dysfunction manifests as attenuated mucus layer, disrupted tight junctions, and paracellular LPS translocation. LPS binding to Toll-like receptor 4 (TLR4) on macrophages activates the MyD88/NF-κB signaling cascade, culminating in pro-inflammatory cytokine release (TNF-α, IL-6) and systemic low-grade inflammation. **Inset**: hepatic FXR activation suppresses CYP7A1 gene transcription, modulating bile acid synthesis. FFAR2/3, free fatty acid receptors 2/3; GLP-1, glucagon-like peptide-1; PYY, peptide YY; HDAC, histone deacetylase; TGR5, Takeda G protein-coupled receptor 5; FXR, farnesoid X receptor; FGF15/19, fibroblast growth factor 15/19; ZO-1, zonula occludens-1; TLR4, Toll-like receptor 4; NF-κB, nuclear factor kappa B.

SCFAs exert protective effects on the intestinal barrier. Butyrate, as the preferred energy source for colonocytes, promotes epithelial cell proliferation and differentiation while enhancing tight junction protein expression (96). Extracellular vesicles secreted by *Akkermansia muciniphila* improve tight junction function through AMPK-dependent mechanisms, reducing intestinal permeability and attenuating high-fat diet-induced weight gain (97). These findings provide novel strategies for improving metabolic diseases through gut microbiota modulation. The molecular signaling networks governing intestinal barrier function operate through distinct mechanisms under homeostatic vs. dysbiotic conditions, with SCFAs and bile acids serving as key regulatory molecules (Figure 2).

## 4 Gut microbiota metabolism of dietary amino acids and its association with diabetic kidney disease

### 4.1 Metabolic interactions between dietary protein and gut microbiota

#### 4.1.1 Effects of protein intake and source on gut microbiota

Dietary protein serves as a crucial nutritional substrate for gut microbiota, with its intake quantity and source significantly influencing intestinal microbial composition and metabolic activity. Patients with diabetic kidney disease (DKD) exhibit pronounced gut microbiota dysbiosis, characterized by decreased abundance of commensal bacteria and increased prevalence of uremic toxin-producing bacteria (7, 12). Tao et al. (98) demonstrated that *Roseburia intestinalis* was significantly decreased while *Bacteroides stercoris* was markedly increased in the gut of biopsy-proven diabetic nephropathy patients, and this dysbiosis was closely correlated with parameters of lipid metabolism, glucose metabolism, and renal function.

Gut microbiota dysbiosis can alter the host metabolome, thereby affecting renal function. Wang et al. (99) demonstrated that aberrant gut microbiota accelerates renal failure progression through host metabolome alterations. In the chronic kidney disease (CKD) state, declining renal function leads to substantial urea excretion into the intestinal tract, where it is hydrolyzed by microbial urease to ammonia, converting to ammonium hydroxide and elevating luminal pH (100). This alkaline environment favors the overgrowth of microorganisms capable of utilizing urea as an energy source while suppressing saccharolytic bacteria that produce short-chain fatty acids (SCFAs) (101). Modulating gut microbiota dysbiosis and improving intestinal barrier function can effectively reduce uremic toxin levels and serum pro-inflammatory mediators, thereby delaying DKD progression (7).

#### 4.1.2 Microbial fermentation metabolism of undigested protein

When protein digestion and small intestinal absorption are incomplete, undigested protein enters the colon for fermentation by gut microbiota (102). The gut microbiota-metabolite axis plays a significant role in early renal function decline, with protein fermentation products closely correlated with renal function parameters (103). Microbial metabolites of aromatic amino acids (tryptophan, tyrosine, and phenylalanine)—phenolic and indolic compounds—undergo sulfation or glucuronidation in the liver before entering systemic circulation, becoming major sources of protein-bound uremic toxins (104). Zhang et al. (105) employed untargeted metabolomics and 16S rRNA gene sequencing to reveal significant alterations in serum metabolites and gut microbiota composition in DKD rats, primarily involving carbohydrate, lipid, and amino acid metabolism, with phenylalanine, tyrosine, and tryptophan metabolic pathways being significantly enriched.

#### 4.1.3 Differences in microbial metabolism between animal and plant proteins

Animal and plant proteins exert distinctly different effects on gut microbiota metabolism. Vegetarian and vegan diets can shift proteolytic gut microbiota toward saccharolytic profiles, reducing uremic toxin production while promoting SCFA generation, enhancing intestinal barrier function, and attenuating inflammation (106). Plant proteins are generally pH-neutral, whereas animal proteins (particularly highly processed meats) exacerbate metabolic acidosis in CKD patients (107). Plant-dominant low-protein diets (PLADO) can delay DKD progression through multiple mechanisms, including reducing dietary acid load, increasing dietary fiber intake, enhancing gut microbiota diversity, and reducing uremic toxin production (108). Restricting intake of potassium-rich fruits and vegetables inhibits the conversion of fermentable dietary fiber to SCFAs, leading to gut microbiota dysbiosis and increased intestinal protein fermentation, thereby increasing the formation of gut-derived uremic toxins (109).

### 4.2 Aromatic amino acid microbial metabolism and uremic toxins

#### 4.2.1 Tryptophan metabolic pathways

##### 4.2.1.1 Generation of indole and its derivatives

Tryptophan is an essential aromatic amino acid, primarily derived from dietary sources including fish, milk, oats, and cheese (7). Dietary tryptophan serves as a precursor for various key metabolites, including kynurenine, serotonin, indole, and indole derivatives (110). In the gastrointestinal tract, tryptophan metabolism proceeds through three main pathways: (1) direct microbial conversion of tryptophan to various aryl hydrocarbon receptor (AhR) ligand molecules, such as indole-3-acetic acid (IAA), indole-3-aldehyde, and indole-3-propionic acid; (2) the kynurenine pathway through the rate-limiting enzyme indoleamine 2,3-dioxygenase (IDO); and (3) the serotonin pathway involving tryptophan hydroxylase-1 in enterochromaffin cells (14). Tryptophan is degraded by bacterial tryptophanase to indole, which participates in maintaining intestinal epithelial tight junction integrity, regulating anti-inflammatory gene expression, and sustaining host-microbiota homeostasis at mucosal surfaces (111).

##### 4.2.1.2 Nephrotoxic mechanisms of indoxyl sulfate (IS)

Indoxyl sulfate (IS) is one of the most representative protein-bound uremic toxins, formed from indole produced by gut microbiota metabolism of tryptophan through hepatic sulfation. Notably, Kikuchi et al. (112) discovered that gut microbiome-derived phenyl sulfate is another important uremic toxin that directly contributes to albuminuria in diabetic kidney disease. IS is approximately 90% bound to albumin in blood circulation and is primarily secreted and excreted through organic anion transporters (OAT1 and OAT3) in renal proximal tubules (113). Due to its high protein-binding characteristics, IS is difficult to remove effectively by dialysis, leading to progressive accumulation as renal function declines (114).

The nephrotoxic mechanisms of IS have been characterized primarily through *in vitro* and animal models, with limited validation in human DKD. Proposed mechanisms include: OAT1/3-mediated cellular uptake inducing apoptosis and necrosis; increased oxidative stress and reduced antioxidant capacity; and TGF-β1 upregulation promoting myofibroblast transformation and fibrosis (115).

Critical interpretive considerations: elevated IS in CKD patients reflects both increased microbial production and impaired renal clearance. Cross-sectional correlations between IS and renal function cannot establish causality. Prospective studies are needed to determine whether IS elevation precedes renal decline or merely reflects reduced GFR. Additionally, IS levels are influenced by dietary protein intake (particularly aromatic amino acids), gut transit time, and medications (phosphate binders, antibiotics). Mechanistic evidence from animal models requires validation in human interventional studies before therapeutic targeting can be recommended.

IS upregulates signal transducer and activator of transcription 3 (STAT3) phosphorylation, increasing production of TGF-β1, monocyte chemoattractant protein-1 (MCP-1), and α-smooth muscle actin (α-SMA), participating in interstitial inflammation and renal fibrosis (116). Lin et al. (117) confirmed that IS severely accumulates in patients with end-stage renal disease (ESRD), accompanied by significant expansion of indole-producing bacteria and upregulation of bacterial tryptophan metabolic pathways.

##### 4.2.1.3 Aryl hydrocarbon receptor (AhR) signaling and renal fibrosis

The aryl hydrocarbon receptor (AhR) is a ligand-activated cytoplasmic transcription factor involved in regulating multiple cellular processes (118). Gut microbiota-derived tryptophan metabolism can mediate renal fibrosis through AhR signaling activation (119). In its resting state, AhR forms a complex with heat shock protein 90 (HSP90), X-associated protein 2 (XAP2), and p23, localized in the cytoplasm. When ligands (such as IS, IAA, and kynurenine) bind to AhR, AhR is released from the chaperone protein complex and translocates to the nucleus, where it heterodimerizes with aryl hydrocarbon receptor nuclear translocator (ARNT), then binds to xenobiotic response elements (XRE) or dioxin response elements (DRE) to induce downstream target gene transcription (120). Research by Xie et al. (121) published in 2024 revealed a novel mechanism by which AhR, as a uremic toxin receptor, promotes renal senescence and fibrosis: IS upregulates and activates AhR in renal tubular epithelial cells, and AhR can interact with peroxisome proliferator-activated receptor gamma coactivator 1-alpha (PGC1α), promoting its ubiquitin-mediated degradation through its E3 ubiquitin ligase activity, thereby inhibiting mitochondrial biogenesis. Notably, ligand-activated AhR may also antagonize TGF-β1 and collagen expression, suggesting that AhR may have dual roles in CKD.

#### 4.2.2 Tyrosine and phenylalanine metabolism

##### 4.2.2.1 Production and hazards of p-cresyl sulfate (pCS)

p-Cresyl sulfate (pCS) is another important protein-bound uremic toxin, formed from p-cresol produced by anaerobic bacterial degradation of tyrosine and phenylalanine through hepatic sulfation or glucuronidation (122). Toft et al. (123) found that the microbial metabolite p-cresol can inhibit gut hormone expression and regulate small intestinal transit, suggesting its important role in intestinal metabolic regulation. The nephrotoxic mechanisms of pCS primarily involve inducing oxidative stress through activation of nicotinamide adenine dinucleotide phosphate (NADPH) oxidase, triggering inflammatory responses, and promoting renal fibrosis (124). Lin et al. (117) found that the relative abundance of p-cresol-producing bacteria was significantly increased in ESRD patients, with markedly elevated serum pCS concentrations. Di Paola et al. (125) noted in their 2023 review that pCS and IS can upregulate nuclear factor-κB (NF-κB) activity, triggering pro-inflammatory processes and increasing reactive oxygen species (ROS) production, and may also play a role in colorectal cancer development and progression in CKD patients.

##### 4.2.2.2 Metabolic significance of phenylacetylglutamine

Phenylacetylglutamine (PAGln) is a gut microbiota-dependent metabolite that has attracted considerable attention in recent years, formed from dietary phenylalanine metabolized by gut microbiota to phenylacetic acid and subsequently conjugated with glutamine in the liver (126). Research by Zhu et al. (127) published in Cell Host Microbe in 2023 identified two distinct gut microbial pathways involved in PAGln production. PAGln is closely associated with atherosclerotic cardiovascular disease (ASCVD) risk, primarily through mechanisms of enhanced platelet reactivity and thrombosis (126). Romano et al. (128) confirmed in 2023 that PAGln is independently associated with heart failure risk, acting through interactions with multiple adrenergic receptors in cardiovascular disease pathogenesis. PAGln is also a common toxin in CKD, significantly accumulating in CKD patients and associated with overall mortality and cardiovascular disease (129), linking gut microbiota metabolism, CKD, and cardiovascular complications.

### 4.3 Branched-chain amino acid (BCAA) metabolism and DKD

#### 4.3.1 Dietary BCAAs and insulin resistance

Branched-chain amino acids (BCAAs), including leucine, isoleucine, and valine, constitute approximately 15%−20% of dietary protein (130). BCAAs play important roles in regulating protein synthesis, primarily through activation of the mammalian target of rapamycin (mTOR) signaling pathway (131). Epidemiological studies consistently demonstrate that elevated circulating BCAA levels are closely associated with increased risk of insulin resistance (IR) and type 2 diabetes mellitus (T2DM) (38). Elevated plasma BCAA levels can appear years before T2DM diagnosis and serve as predictive biomarkers for IR and T2DM (132, 133). Regarding the causal relationship between BCAAs and IR, two main hypotheses currently exist: sustained high BCAA levels interfere with insulin signaling through mTOR complex 1 (mTORC1) activation leading to insulin receptor substrate-1 (IRS-1) serine phosphorylation; BCAA catabolic defects lead to accumulation of metabolic intermediates, causing mitochondrial dysfunction and oxidative stress (133). Zhenyukh et al. (134) confirmed that high concentrations of BCAAs promote oxidative stress, inflammation, and migration in human peripheral blood mononuclear cells through mTORC1 activation.

#### 4.3.2 BCAAs-mTOR signaling and glomerular injury

The interaction between BCAAs and the mTOR signaling pathway plays an important role in DKD glomerular injury. Li et al. (135) noted in their 2023 review that BCAA overload can activate the mTOR pathway while inhibiting the phosphatidylinositol 3-kinase (PI3K)-protein kinase B (Akt) intracellular pathway, leading to IR. mTOR is also a key factor inhibiting autophagy in renal tubular epithelial cells, and leucine may regulate cellular autophagy through the mTOR pathway to attenuate renal injury. Zhu et al. (136) published research in 2022 demonstrating that DKD progression in T2DM patients is associated with impaired amino acid metabolism. Tanase et al. (137) further indicated that DKD microvascular complications are closely associated with abnormal BCAA metabolism, with high levels of L-leucine [area under the curve (AUC) = 0.834] and isoleucine (AUC = 0.932) demonstrating high diagnostic ability in distinguishing DKD from T2DM.

#### 4.3.3 Microbial regulation of BCAA metabolism

Gut microbiota plays an important regulatory role in BCAA metabolism. Kim et al. (138) published research in Microbiology Spectrum in 2023 finding that methionine, leucine, isoleucine, and valine levels were significantly elevated while acetate levels were decreased in DKD patients. Random forest model analysis showed that methionine and BCAAs (AUC = 0.832) were the most significant features distinguishing DKD patients from healthy controls. Tao et al. (98) found that BCAA biosynthesis was significantly reduced in gut microbiota functional analysis of DKD patients. Liu et al. (139) confirmed in 2023 that serum BCAA levels can serve as effective indicators of DKD, with BCAA levels beginning to decline in the microalbuminuria stage and further declining in the macroalbuminuria stage, negatively correlated with urinary microalbumin levels and positively correlated with serum albumin levels, indicating that BCAAs are closely related to nutritional status in T2DM patients.

### 4.4 Sulfur-containing amino acid metabolism and renal protection

#### 4.4.1 Metabolic fate of methionine and cysteine

Sulfur-containing amino acids (methionine and cysteine) play critical roles in maintaining redox balance and producing protective gaseous signaling molecules (140). Methionine is an essential amino acid that is converted to cysteine through the transsulfuration pathway, which further participates in glutathione synthesis and hydrogen sulfide (H_2_S) generation. Kim et al. (138) showed that methionine metabolism gene expression was significantly altered in the gut microbiota of DKD patients. Chen et al. (141) found in 2023 that renal cystathionine γ-lyase (CSE) expression was significantly reduced in diabetic mouse models, potentially leading to decreased H_2_S generation and consequently exacerbating renal injury.

#### 4.4.2 Renoprotective effects of hydrogen sulfide (H_2_S)

Hydrogen sulfide (H_2_S) is the third gaseous signaling molecule after nitric oxide and carbon monoxide, playing important roles in renal physiological and pathological processes (142). H_2_S can improve diabetic renal injury through multiple mechanisms: alleviating high glucose-induced oxidative stress and activating the nuclear factor erythroid 2-related factor 2 (Nrf2) antioxidant pathway; exerting anti-inflammatory effects by inhibiting NF-κB signaling; reducing glomerular mesangial cell proliferation by blocking the mitogen-activated protein kinase (MAPK) signaling pathway; and inhibiting renin-angiotensin system activity (143). Chen et al. (141) further confirmed in 2023 that the H_2_S donor GYY4137 improves renal oxidative damage in diabetic mice by decreasing NADPH oxidase 2 (NOX2) expression and increasing heme oxygenase-2 (HO-2) and paraoxonase 1/2 (PON1/2) expression. However, H_2_S has dual effects in the kidney and may also mediate renal injury in certain disease models, suggesting its context-dependent actions (144).

#### 4.4.3 Homocysteine and DKD vascular injury

**Figure 3:**
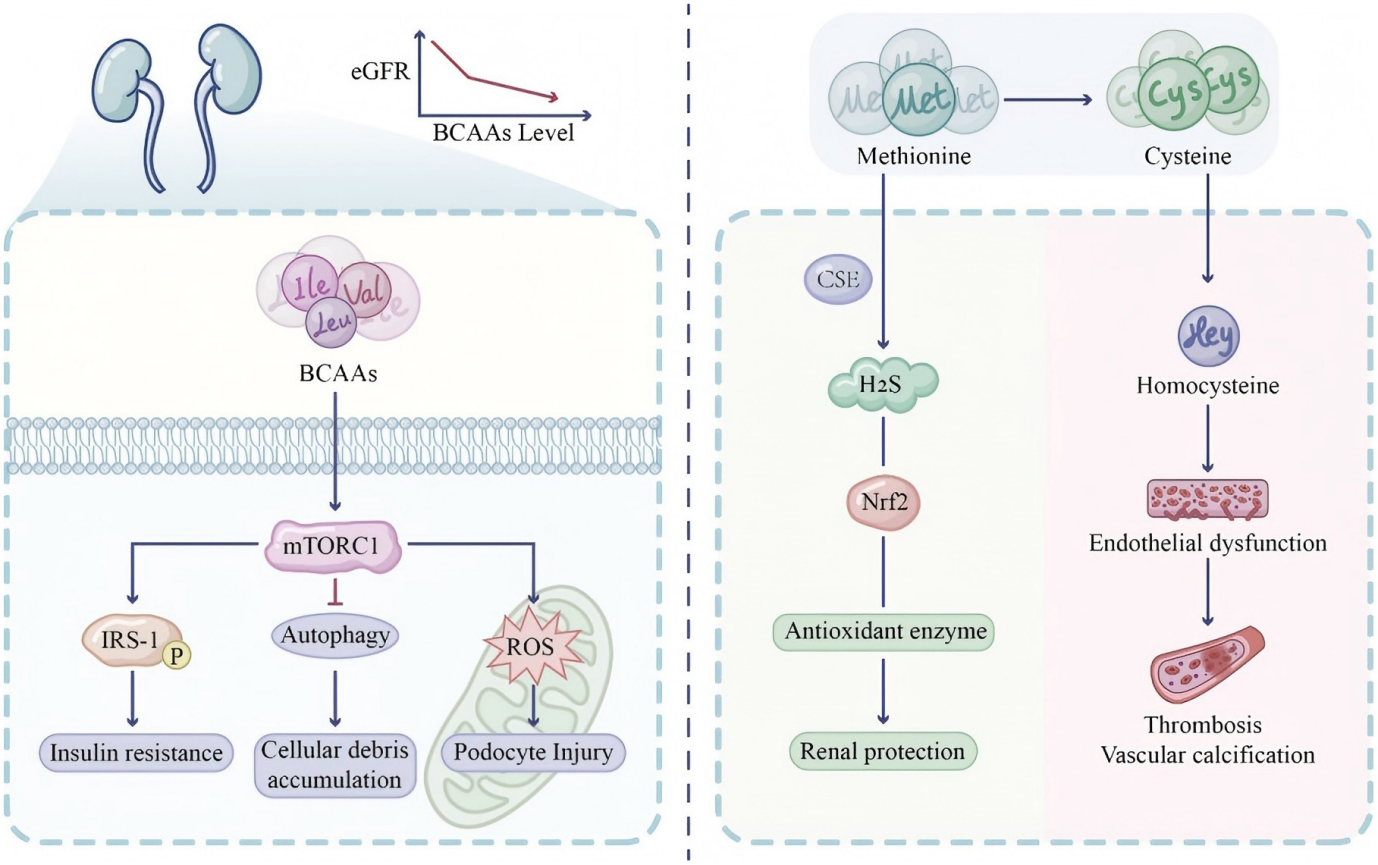
Amino acid signaling pathways: the dual effects of branched-chain amino acids and sulfur-containing amino acids in metabolic regulation. This dual-panel illustration demonstrates the paradoxical effects of amino acid metabolism in renal pathophysiology. **Panel A (BCAAs and mTOR signaling)**: elevated circulating branched-chain amino acids (BCAAs: leucine, isoleucine, valine) activate the mechanistic target of rapamycin complex 1 (mTORC1), depicted as a central signaling hub. Chronic mTORC1 hyperactivation induces insulin receptor substrate-1 (IRS-1) serine phosphorylation, thereby attenuating insulin signaling and promoting insulin resistance. Simultaneously, mTORC1 suppresses autophagy, leading to cellular waste accumulation, while oxidative stress propagates podocyte injury. **Inset graph**: correlation between serum BCAA concentrations and estimated glomerular filtration rate (eGFR) decline. **Panel B (sulfur amino acid metabolism)**: methionine-to-cysteine conversion bifurcates into two distinct pathways. **Protective pathway**: cystathionine γ-lyase (CSE) catalyzes hydrogen sulfide (H_2_S) production, which activates nuclear factor erythroid 2-related factor 2 (Nrf2), inducing antioxidant enzyme expression (HO-1) and conferring renoprotection. CSE expression is diminished in diabetic kidney disease (DKD). **Deleterious pathway**: homocysteine (Hcy) accumulation promotes endothelial dysfunction, vascular calcification, and thrombotic risk. Color coding: red/orange indicates pathological hyperactivation; cyan/blue represents protective antioxidant responses. BCAAs, branched-chain amino acids; mTORC1, mechanistic target of rapamycin complex 1; IRS-1, insulin receptor substrate-1; CSE, cystathionine γ-lyase; H_2_S, hydrogen sulfide; Nrf2, nuclear factor erythroid 2-related factor 2; HO-1, heme oxygenase-1; Hcy, homocysteine.

Homocysteine (Hcy) is an intermediate metabolite formed during the conversion of methionine to cysteine, and elevated plasma levels (hyperhomocysteinemia, HHcy) are closely associated with increased cardiovascular disease risk (145). Hcy can damage endothelial cells through multiple pathways, including oxidative stress, endoplasmic reticulum stress, inhibition of nitric oxide (NO) production, and induction of inflammatory responses (146). HHcy is extremely common in DKD patients, with prevalence increasing as renal function declines. Shen et al. (147) analyzed National Health and Nutrition Examination Survey (NHANES) data and found that HHcy was positively associated with CKD risk [odds ratio (OR) 1.17; 95% confidence interval (CI) 1.11–1.22]. Alsolami et al. (148) noted in their 2023 systematic review that the prevalence of HHcy in ESRD patients is approximately 85%−100%, significantly increasing cardiovascular event risk. Amino acid metabolism in DKD exhibits paradoxical effects: while BCAAs activate mTORC1 signaling leading to insulin resistance and podocyte injury, sulfur amino acid-derived H_2_S confers renoprotection through Nrf2 activation, whereas homocysteine accumulation promotes vascular damage (Figure 3). Table 1 summarizes the role of amino acid metabolism in DKD, covering microbial pathways, uremic toxins, and renal effects.

**Table 1:** Amino acid metabolism in DKD: microbial pathways, uremic toxins, and renal consequences.

Amino acid category	Representative amino acids	Key gut microbial genera	Microbial enzymes	Primary metabolites	Hepatic conjugation	Renal effects	Molecular mechanisms	Evidence level	Key references
Aromatic amino acids (AAA)									
Tryptophan	L-Tryptophan	*Clostridium sporogenes, Bacteroides, Escherichia coli*	Tryptophanase (TnaA)	Indole → **Indoxyl sulfate (IS)**	Sulfotransferase (SULT1A1)	↑ Oxidative stress, ↑ Fibrosis, ↑ Inflammation	•OAT1/3 uptake → ↑ ROS/TGF-β1 •AhR activation → PGC1α degradation → ↓ mitochondrial biogenesis	Human observational + Animal models	(112, 115, 116, 121)
		*Lactobacillus, Bifidobacterium* (↓ in DKD)	Indole-3-pyruvate pathway	Indole-3-propionic acid (protective)	None (circulates unchanged)	↑ Intestinal barrier integrity	• AhR activation → ↑ tight junctions	Animal models	(14, 111)
Tyrosine/Phenylalanine	L-Tyrosine, L-Phenylalanine	*Clostridium difficile, Bacteroides fragilis*	Tyrosine decarboxylase, Phenylalanine deaminase	p-Cresol → **p-Cresyl sulfate (pCS)**	Sulfation (SULT1A1)/Glucuronidation	↑ Oxidative stress, ↑ Inflammation, ↑ Fibrosis	•NADPH oxidase activation → ↑ ROS •↑ NF-κB → pro-inflammatory cytokines	Human observational + Animal models	(117, 122, 124, 125)
Phenylalanine	L-Phenylalanine	*Clostridium, Eubacterium*	Phenylacetate dehydrogenase	Phenylacetic acid → **Phenylacetylglutamine (PAGln)**	Glutamine conjugation (hepatic)	↑ Platelet reactivity, ↑ Thrombosis, ↑ CVD risk	•Adrenergic receptor activation (α2A, α2B, β2)	Human mechanistic + Observational	(126–129)
Branched-chain amino acids (BCAA)									
Leucine, Isoleucine, Valine	L-Leucine, L-Isoleucine, L-Valine	*Gemmiger* (↑ in DKD), *Prevotella, Bacteroides*	Branched-chain aminotransferase, Branched-chain α-keto acid dehydrogenase	Elevated circulating BCAAs (↓ microbial biosynthesis paradox)	Not applicable (amino acids, not conjugated)	↑ Insulin resistance, ↑ Podocyte injury, ↓ Autophagy	•mTORC1 activation → IRS-1 Ser phosphorylation → ↓ insulin signaling •↓ Autophagy → cellular waste accumulation	Human observational + Animal models	(134, 136–138, 152)
Sulfur-containing amino acids (SAA)									
Methionine	L-Methionine	*Bacteroides, Clostridium, Desulfovibrio*	Methionine γ-lyase	**Hydrogen sulfide (H**_**2**_**S)** (via transsulfuration: Met → Cys → H_2_S)	None (gaseous signaling molecule)	↓ Oxidative stress, ↓ Inflammation, ↓ Mesangial proliferation	•Nrf2 activation → ↑ HO-1/PON1/2 •↓ NF-κB inflammatory signaling •↓ MAPK proliferation pathway	Animal models	(141–144)
Methionine (Aberrant pathway)	L-Methionine	Dysbiosis → ↓ CSE expression	Impaired transsulfuration	**Homocysteine (Hcy)** (accumulation)	Not applicable (intermediate metabolite)	↑ Endothelial dysfunction, ↑ Vascular calcification, ↑ CVD risk	•Oxidative stress + ER stress •↓ NO production •Pro-thrombotic state	Human observational	(145–148)

Dysbiosis signature in DKD: ↓ SCFA-producers (Roseburia intestinalis, Faecalibacterium prausnitzii), ↑ Proteobacteria (Escherichia-Shigella), ↑ Bacteroides stercoris, ↑ Gemmiger (169, 190). Evidence level key: (1) Human observational: Cohort/cross-sectional studies with metabolomic/microbiome profiling; (2) Animal models: Primarily db/db mice, STZ-induced diabetes, high-fat diet models; and (3) Human mechanistic: *In vitro* studies with human cells or ex vivo patient samples. Bold values indicate the most clinically significant gut microbiota-derived uremic toxins and their associated microbial genera, which have been validated in human observational studies or human mechanistic studies (as specified in the Evidence Level column). These entries represent the highest-priority therapeutic targets within the gut microbiota–kidney axis in DKD, given their demonstrated accumulation in patients with declining renal function and their direct association with oxidative stress, inflammation, and fibrosis. Non-bolded entries reflect metabolites and pathways supported primarily by animal model evidence, where causal roles in human DKD remain to be prospectively validated.

#### 4.4.4 Integrative perspective: convergent and divergent metabolic fates of amino acids in DKD

**Figure 4:**
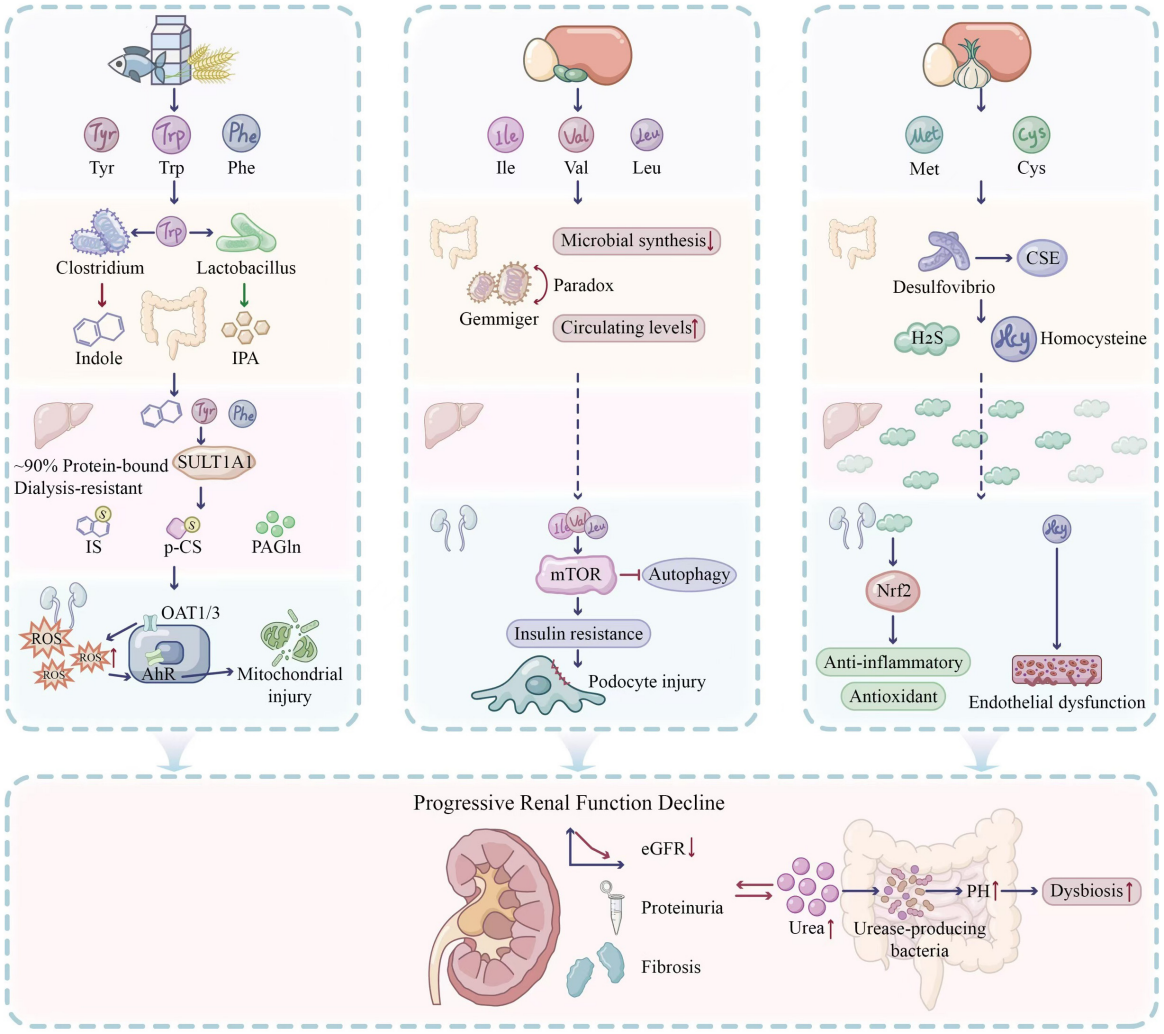
Landscape of Gut-Liver-Kidney Axis-Mediated Amino Acid Metabolic Disorders in Diabetic Kidney Disease. The figure illustrates the distinct pathophysiological trajectories of three major amino acid groups—Aromatic (AAA), Branched-chain (BCAA), and Sulfur-containing (SAA) amino acids—as they traverse the gut, liver, and kidney, culminating in progressive renal decline. **Left panel (AAA)**: dietary Trp, Tyr, and Phe are fermented by gut bacteria (e.g., *Clostridium*) into precursors like Indole. In the liver, these are conjugated by SULT1A1 into protein-bound uremic toxins (IS, pCS), which are poorly cleared by dialysis. Upon entering proximal tubular cells via OAT1/3, they activate the Aryl Hydrocarbon Receptor (AhR), triggering mitochondrial injury and ROS production. **Middle panel (BCAA)**: illustrates the “BCAA Paradox” where circulating levels are elevated despite reduced microbial synthesis. Excess BCAAs bypass hepatic processing and hyperactivate mTORC1 in podocytes, inhibiting autophagy and inducing insulin resistance and cellular injury. **Right panel (SAA)**: depicts the dual nature of sulfur metabolism. While Homocysteine drives endothelial dysfunction, microbial-derived H_2_S (via *Desulfovibrio*) offers protective effects by activating the Nrf2 antioxidant pathway. **Bottom panel**: the convergence of these pathways leads to progressive renal function decline (proteinuria, fibrosis). The resulting accumulation of urea feeds back into the gut, increasing pH and exacerbating dysbiosis (The Vicious Cycle).

The preceding sections delineate distinct metabolic trajectories for aromatic amino acids (AAA), branched-chain amino acids (BCAA), and sulfur-containing amino acids (SAA) within the gut-liver-kidney axis. However, a unified perspective reveals both convergent pathophysiological endpoints and paradoxical protective pathways that collectively define the metabolic landscape of DKD (see Figure 4).

##### 4.4.4.1 Convergent nephrotoxic mechanisms

Despite originating from disparate amino acid precursors, protein-bound uremic toxins (IS, pCS, PAGln) share common renal injury mechanisms. All three are actively transported into proximal tubular cells via organic anion transporters (OAT1/3), where they trigger oxidative stress through NADPH oxidase activation and reactive oxygen species (ROS) generation (115, 124). Simultaneously, BCAA-mediated mTORC1 hyperactivation in podocytes suppresses autophagy and exacerbates insulin resistance, creating a metabolic milieu that potentiates toxin-induced injury (136, 149). The cumulative effect is a self-reinforcing cycle: declining renal function → impaired toxin clearance → progressive tubular and glomerular damage → further GFR decline.

##### 4.4.4.2 Gut microbiota dysbiosis as the central driver

A unifying feature across AAA, BCAA, and SAA metabolism is the pathogenic role of gut microbiota dysbiosis in DKD. In the SAA pathway, dysbiosis-associated inflammation downregulates renal cystathionine γ-lyase (CSE), shunting methionine metabolism toward homocysteine accumulation rather than protective H_2_S generation (141).

##### 4.4.4.3 Paradoxical protective pathways within the same metabolic network

Not all amino acid-derived metabolites are deleterious. Tryptophan metabolism bifurcates into both toxic (IS via tryptophanase) and protective (indole-3-propionic acid via alternative pathways) branches, with the latter enhancing intestinal barrier integrity through AhR-mediated tight junction upregulation (14, 111). Similarly, the SAA pathway generates H_2_S—a gaseous signaling molecule that activates Nrf2 antioxidant defenses, suppresses NF-κB inflammation, and inhibits mesangial cell proliferation (142, 143). This duality underscores the critical importance of microbial community composition over total amino acid load: a eubiotic microbiome channels tryptophan and methionine toward protective metabolites, whereas dysbiosis skews production toward nephrotoxins.

##### 4.4.4.4 Clinical implications: precision targeting of amino acid metabolism

These mechanistic insights inform precision nutritional strategies:

1. Plant protein prioritization reduces aromatic amino acid fermentation to uremic toxins while increasing fiber availability for SCFA production (106, 108).
2. Prebiotic supplementation (inulin, resistant starch) selectively enriches SCFA-producers, potentially rebalancing tryptophan metabolism toward protective indole derivatives (150, 151).
3. H_2_S donors (e.g., GYY4137) may bypass dysbiosis-impaired endogenous synthesis, directly conferring renoprotection (141).
4. BCAA restriction in advanced DKD may mitigate mTORC1-driven podocyte injury, though this requires validation against risks of protein-energy wasting (135).

##### 4.4.4.5 Biomarker integration for disease staging and intervention monitoring

The metabolite panel encompassing IS, pCS, PAGln, circulating BCAAs, and homocysteine provides a comprehensive readout of gut-liver-kidney axis dysregulation. Machine learning models integrating these metabolites with gut microbiota profiles (e.g., Gemmiger abundance, SCFA-producer/proteolytic bacteria ratios) demonstrate superior DKD risk stratification compared to traditional markers alone (AUC 0.83–0.93) (136, 138, 152). Longitudinal monitoring of this panel during dietary or probiotic interventions can objectively assess gut microbiota modulation efficacy, guiding personalized treatment optimization.

In summary, amino acid metabolism in DKD is not a collection of isolated pathways but an interconnected network where gut microbiota composition determines the balance between nephrotoxic and nephroprotective outputs. Therapeutic strategies must therefore target the microbial ecosystem holistically rather than restricting individual amino acids, leveraging the metabolic plasticity of the gut-liver-kidney axis to shift metabolic flux toward renoprotection.

### 4.5 Potential of amino acid metabolites as nutritional biomarkers for DKD

#### 4.5.1 Key biomarkers identified through metabolomics

The development of metabolomics technology has provided powerful tools for DKD biomarker discovery. Zhu et al. (136) confirmed in 2022 that amino acid metabolism plays an important role in DKD progression. Through metabolomic analysis of T2DM and DKD patients, they found that N-acetylaspartate, L-valine, betaine, isoleucine, asparagine, and L-methionine were significantly elevated in DKD patients, with high levels of L-leucine and isoleucine significantly associated with rapid estimated glomerular filtration rate (eGFR) decline. Roointan et al. (153) identified lactate, hippuric acid, urea, and glutamine as the most important non-invasive early diagnostic biomarkers through meta-analysis.

#### 4.5.2 Nutritional metabolic signature profiles for predicting disease progression

Establishing nutritional metabolic signature profiles for predicting DKD progression is an important current research direction. Machine learning-assisted metabolomics analysis provides new possibilities for this endeavor (152). The diagnostic value of specific amino acid metabolic markers has been preliminarily validated. Ding et al. (154) evaluated the value of combined serum Hcy and neuregulin 4 (NRG4) measurement in early diagnosis of DKD complicating T2DM in 2023. Two oligopeptides (Asn-Met-Cys-Ser and Asn-Cys-Pro-Pro) were associated with proteinuria severity, with AUC values of 0.8857 and 0.9963, respectively (12). Future research should focus on establishing comprehensive assessment systems containing multiple amino acid metabolites, validating the universality of metabolic markers across different populations, exploring longitudinal associations between metabolic markers and clinical outcomes, and developing personalized nutritional intervention strategies based on metabolomics.

#### 4.5.3 Methodological considerations in biomarker research

##### 4.5.3.1 Evidence strength assessment and study limitations

Current metabolomic investigations predominantly employ cross-sectional designs or case-control methodologies, which harbor intrinsic deficiencies in causal inference. Elevated plasma concentrations of IS and pCS may arise through two distinct mechanisms: impaired clearance secondary to compromised glomerular filtration, or pathological triggering events during early disease phases (155). This bidirectional nature of causal relationships constitutes a primary obstacle in the clinical interpretation of metabolomic data.

##### 4.5.3.2 Principal factors influencing biomarker application

**Impact of renal functional status**: when glomerular filtration rate declines, multiple metabolic intermediates accumulate systemically, including branched-chain amino acids and aromatic amino acid catabolites, rendering differentiation between authentic pathogenic markers and consequences of diminished renal clearance capacity difficult (50, 51). The critical question lies in determining whether elevated circulating metabolite concentrations represent disease-driving factors or secondary manifestations of excretory dysfunction.

**Temporal dependence of nutritional intake**: short-term dietary composition adjustments can rapidly alter amino acid profiles in serum and urine (31). The regulatory effects of macronutrient ratios on intestinal microecological systems and metabolite biosynthetic pathways manifest within days (31), introducing substantial intra-individual variability and inter-individual differences that confound biomarker stability interpretation.

**Inter-individual microecological variations**: gut microbiota compositional architecture exhibits 2–3-fold fluctuations across different timepoints within individual subjects and among different individuals (28). These variations are predominantly determined by external environmental factors rather than host genetic background (28), limiting both metabolite measurement reproducibility and clinical application reliability of biomarkers.

**Compound effects of pharmacological therapy**: standard treatment regimens for DKD patients incorporate sodium-glucose cotransporter 2 inhibitors and renin-angiotensin system inhibitors, which exert influence through multiple pathways including intestinal pH environment modification, membrane transporter expression regulation, and metabolomic landscape remodeling (156, 157). The demonstrated restructuring effects of SGLT2 inhibitors on gut microbial community architecture and their regulatory actions on metabolite generation (156) may confound the authenticity of associations between metabolites and disease.

##### 4.5.3.3 Current validation framework status

Although numerous studies employ area under receiver operating characteristic curves exceeding 0.8 as diagnostic performance criteria, validation data from independent external cohorts remain insufficient. Clinical translation requires the following elements: cross-regional, multicenter validation in at least three independent cohorts with population heterogeneity; establishment of longitudinal follow-up systems capable of assessing predictive efficacy beyond mere diagnostic discrimination; monitoring of biomarker dynamic responsiveness during therapeutic interventions; completion of health economic evaluation and clinical feasibility demonstration (158).

##### 4.5.3.4 From correlation to causation

Establishing causal linkage between amino acid metabolites and DKD necessitates integration of evidence from diverse research domains.

At the epidemiological observation level, prospective cohort studies have confirmed the predictive value of baseline metabolite concentrations for subsequent disease progression, with current evidence strength graded as moderate (159). At the genetic epidemiology level, Mendelian randomization-based analyses provide support for causal relationship hypotheses, though evidence strength remains preliminary (155). At the pathological mechanism level, animal model experiments have verified direct nephrotoxic effects of metabolites, with relatively substantial evidence in this domain (102, 112). At the clinical intervention level, evidence regarding whether reducing specific metabolite levels can improve patient clinical outcomes remains deficient (160).

Synthesizing available evidence, IS and pCS possess relatively complete evidence chains (encompassing Levels 1–3 evidence), whereas PAGln and certain BCAA derivatives require additional empirical research for verification.

##### 4.5.3.5 Future perspectives in research pathways

Establishment of large-scale longitudinal tracking platforms: international collaborative cohorts enrolling >5,000 DKD patients must be assembled to conduct 5–10-year systematic tracking observations, clarifying temporal associations between hard endpoint events (end-stage renal disease, all-cause mortality) and metabolites. Study protocols should incorporate standardized dietary documentation, medication usage trajectory tracking, and continuous dynamic metabolite monitoring to control time-dependent confounding factors (161). Integrated analysis of multi-omics data: integration of metagenomic sequencing technology, untargeted metabolomics analysis, and detailed clinical phenotype data should construct systems biology network models of microecology-metabolite-disease interactions (162). Comprehensive analysis of gut microbial community structural characteristics, metabolite characteristic profiles, and clinical phenotype information promises discovery of critical pathogenic pathways and potential intervention targets (163) Targeted metabolic pathway intervention trials: randomized controlled clinical studies directed at specific metabolic pathways should be designed. Research strategies may include employing oral adsorbent materials AST-120 to remove IS and pCS (160, 164), or utilizing prebiotic preparations to modulate gut microbiota composition (20), thereby evaluating the inhibitory effects of metabolite burden reduction strategies on DKD progression. Trial designs require rigorous dietary standardization measures and comprehensive safety monitoring mechanisms. Precision stratification based on metabolic phenotypes: DKD patients should be categorized into distinct metabolic subgroups according to characteristic amino acid metabolic patterns, such as “IS high-burden phenotype” or “BCAA metabolic imbalance phenotype,” with exploration of specific nutritional medicine intervention strategies for each subgroup (165). This approach aligns with core concepts of precision nutrition (31), enabling identification of patient populations exhibiting optimal responsiveness to specific intervention modalities. Standardization process for detection technologies: international unified standards for metabolite quantitative detection must be formulated, encompassing preparation of standard reference materials, analytical methods for detection instrumentation, and operational protocols for quality control, to enhance comparability among results from different studies (166). Achieving standardization across the entire workflow from sample collection and preservation to analytical determination constitutes a necessary prerequisite for translating metabolomic research findings into clinical diagnostic and therapeutic decision-making tools.

Through systematic integration of the aforementioned research directions, amino acid metabolite biomarkers can advance from purely associative discoveries to validated clinical utility tools, ultimately serving individualized nutritional therapy and management of DKD patients.

#### 4.5.4 Methodological limitations and confounding factors in metabolomic biomarker interpretation

While amino acid-derived metabolites show promise as DKD biomarkers, rigorous interpretation requires acknowledging substantial methodological limitations.

##### 4.5.4.1 Study design constraints and causality

Most metabolomic studies linking uremic toxins (IS, pCS, PAGln) and BCAAs to DKD employ cross-sectional or case-control designs. These demonstrate associations but cannot establish causation. For IS correlating with declining eGFR (117), two scenarios exist: (1) Reverse causation—reduced GFR impairs IS clearance, elevating levels independent of pathogenic effects; and (2) Forward causation (unproven in humans)—IS drives tubular injury. Distinguishing these requires prospective cohort studies with pre-decline metabolite measurements and Mendelian randomization analyses using genetic variants in microbial tryptophanase as instrumental variables.

##### 4.5.4.2 Renal function as dominant confounder

Metabolite concentrations are profoundly GFR-dependent, confounding their interpretation as disease-specific biomarkers. Protein-bound uremic toxins (IS, pCS, PAGln) accumulate exponentially as eGFR declines below 60 mL/min/1.73 m^2^ due to saturable OAT1/3-mediated tubular secretion (113, 167), with patients at eGFR 30 exhibiting 5–10 × higher IS than those at eGFR 90—reflecting clearance impairment rather than unique pathogenic mechanisms. Similarly, BCAA elevation in DKD reflects both impaired renal catabolism (reduced mitochondrial BCKDH activity) and insulin resistance-driven alterations (133), with standard eGFR adjustment being insufficient as GFR lies on the causal pathway between metabolites and outcomes.

##### 4.5.4.3 Dietary intake variability

Amino acid metabolites exhibit marked short-term fluctuations driven by dietary intake. A single high-protein meal containing 2–3 g tryptophan can transiently elevate serum IS by 30%−50% within 6–12 h, with fasting status, protein source (animal vs. plant), and recent antibiotic use introducing substantial variability. BCAAs show similar patterns, as dietary intake (8–12 g/day in typical Western diets) directly influences circulating levels with peak concentrations occurring 2–3 h postprandially. Essential mitigation strategies include fasting morning blood draws, 24-h urine collections to integrate metabolite production over diurnal cycles, dietary adjustment in statistical models, and repeated measurements to capture intra-individual variability.

##### 4.5.4.4 Gut microbiota variability

Gut microbiota composition exhibits both high inter-individual variability (explaining 40%−60% of metabolite variance) and significant temporal instability. Enterotype differences (Bacteroides vs. Prevotella vs. Ruminococcus-dominant) fundamentally alter amino acid fermentation efficiency (28), such that two individuals consuming identical diets may produce 3–5 × different IS levels based on *Clostridium* abundance. Moreover, microbiota composition can shift within 24–48 h following dietary changes (31), antibiotic exposure, or gastrointestinal illness, introducing substantial temporal noise. Consequently, single-timepoint IS measurements reflect only a snapshot of an individual's current microbiota state, diet, and renal function rather than a stable metabolic phenotype. Serial sampling (e.g., monthly over 6 months) with concurrent 16S rRNA microbiome profiling is needed to establish whether metabolite elevations are persistent or transient.

##### 4.5.4.5 Pharmaceutical confounding

Common DKD medications substantially alter metabolomic profiles through diverse mechanisms. RAAS inhibitors may reduce proteinuria-associated amino acid losses, indirectly affecting circulating BCAA pools, while SGLT2 inhibitors shift substrate utilization toward ketogenesis and fatty acid oxidation, potentially reducing BCAA oxidation. Antibiotics profoundly disrupt gut microbiota, transiently reducing IS/pCS production for weeks post-treatment, and phosphate binders such as sevelamer bind IS in the gut, lowering circulating levels independent of microbial production changes. Comprehensive medication inventories with dosage and duration should be mandatory covariates in biomarker discovery studies.

##### 4.5.4.6 Recommendations for future studies

To advance amino acid metabolites from associative biomarkers to validated therapeutic targets, the field must prioritize: (1) prospective cohort designs with serial metabolite measurements predating renal function decline; (2) Mendelian randomization using genetic variants in microbial enzyme genes (tryptophanase, tyrosine decarboxylase) or host transporter genes (OAT1/3) as instrumental variables; (3) intervention trials testing metabolite-lowering strategies (prebiotics, AST-120 adsorbents, dietary protein modification) with hard renal outcomes (eGFR slope, ESRD, mortality); (4) multi-omics integration combining metabolomics with metagenomics, transcriptomics, and proteomics to build mechanistic causal models; and (5) standardization protocols for sample collection, processing, and analytical methods to enable meta-analysis.

**Current evidence grade**: based on GRADE criteria, amino acid metabolites merit “Low to Moderate” quality evidence: consistent associations and mechanistic plausibility are offset by predominant cross-sectional designs, high confounding risk, and lack of interventional validation. Clinical adoption should await prospective validation demonstrating improved risk prediction beyond standard parameters (eGFR, albuminuria, HbA1c).

## 5 The gut-liver-kidney axis: a pathological network of nutritional metabolic disorders in diabetic kidney disease

### 5.1 Molecular basis of inter-organ metabolic crosstalk

#### 5.1.1 Systemic circulation of gut-derived metabolites

Research on the pathogenesis of diabetic kidney disease (DKD) has shifted from a single-organ perspective to an integrated view of multi-organ network regulation. The gut-liver-kidney axis theory provides a systematic framework for understanding the complex nutritional metabolic disorders in DKD (7, 168). As the largest metabolic organ in the human body, the gut microbiota composition and its metabolites profoundly influence distant organ function through systemic circulation. Wu et al. (152) employed machine learning-assisted multi-omics analysis to reveal that serum branched-chain amino acid (BCAA) levels were significantly elevated in DKD patients, closely associated with gut microbiota functional reprogramming. Functional prediction analysis identified that the DKD-specific biomarker genus *Gemmiger* was significantly enriched in carbohydrate metabolism and BCAA biosynthesis pathways (152).

The accumulation and impaired clearance of uremic toxins represent the core manifestation of bidirectional gut-kidney axis regulation. When estimated glomerular filtration rate (eGFR) declines, circulating uremic toxins accumulate and reciprocally affect gut microbiota composition and function (169). Gut-derived uremic toxins including indoxyl sulfate (IS), p-cresyl sulfate (pCS), and trimethylamine N-oxide (TMAO) are significantly elevated in DKD patients (6). The metabolic pathway of TMAO involves multi-organ coordination: dietary choline, carnitine, and other quaternary ammonium-containing compounds are metabolized by gut microbiota to produce trimethylamine (TMA), which is subsequently oxidized to TMAO by hepatic flavin-containing monooxygenases (FMOs) and primarily cleared by the kidneys (170). In DKD patients, plasma TMAO concentration is independently associated with cardiovascular mortality risk (171).

Fang et al. (172) demonstrated in diabetic rat models that TMAO treatment exacerbated renal inflammation and fibrosis through activation of the NLRP3 inflammasome, promoting IL-1β and IL-18 release and increasing pro-fibrotic TGF-β1 expression. Furthermore, TMAO can downregulate intestinal tight junction protein expression, increasing intestinal permeability and establishing a vicious cycle of gut leakage and renal injury (173). This gut leakage phenotype, driven by hyperglycemia, uremia, and microbial toxins, is particularly prominent in DKD.

#### 5.1.2 Coordination between hepatic detoxification and renal excretion

The liver, serving as the central node of the gut-liver-kidney axis, plays a critical role in DKD progression through its metabolic dysfunction. A significant epidemiological association exists between metabolic dysfunction-associated steatotic liver disease (MASLD) and DKD (174, 175). Wei et al. (176) conducted a 10-year randomized cohort study and found that type 2 diabetes patients with MASLD had a 46% increased risk of developing CKD (HR 1.46, 95% CI 1.26–1.70). Stratified analysis further revealed that patients aged < 60 years faced higher MASLD-associated CKD risk, emphasizing the importance of early intervention.

Hepatorenal coordination involves complex molecular dialogue networks. Moncho et al. (177) systematically reviewed the interactions among MASLD, CKD, and cardiovascular disease, highlighting insulin resistance, chronic inflammation, and progressive fibrosis as key mediating factors. Liver-secreted hepatokines regulate distant organ function through endocrine pathways (178). Fibroblast growth factor 21 (FGF-21), primarily synthesized in the liver, enhances hepatic insulin sensitivity, reduces hepatic triglyceride accumulation, and possesses anti-inflammatory, antioxidant, and anti-fibrotic properties (179, 180). Chen et al. (181) revealed the central role of the renal UTX-PHGDH-serine axis in simultaneously regulating metabolic disorders in both kidneys and liver.

The CArdio-Renal-DIAbetes-Liver-Metabolic Syndrome (CARDIAL-MS) concept proposed by Godoy-Matos et al. (182) systematized this multi-organ interaction. This model integrates MASLD as a key component of metabolic syndrome, encompassing four progressive stages: weight gain and adipose tissue dysfunction, metabolic risk factors, cardiometabolic disease and CKD, and advanced cardiorenal-hepatic metabolic disease. This staging model helps identify critical nodes in the transition from reversible metabolic disorders to irreversible organ damage.

#### 5.1.3 Metabolic waste accumulation and multi-organ damage

Amino acid metabolic disorders are important features of multi-organ damage in DKD. Zhu et al. (136) identified through metabolomic studies that serum N-acetylaspartate, L-valine, betaine, and isoleucine were significantly upregulated in DKD patients. Random forest model analysis demonstrated that methionine and branched-chain amino acids (AUC = 0.832) were the most important features distinguishing DKD patients from healthy controls (138). L-leucine (AUC = 0.834) and isoleucine (AUC = 0.932) levels were significantly associated with rapid eGFR decline (136).

**Figure 5:**
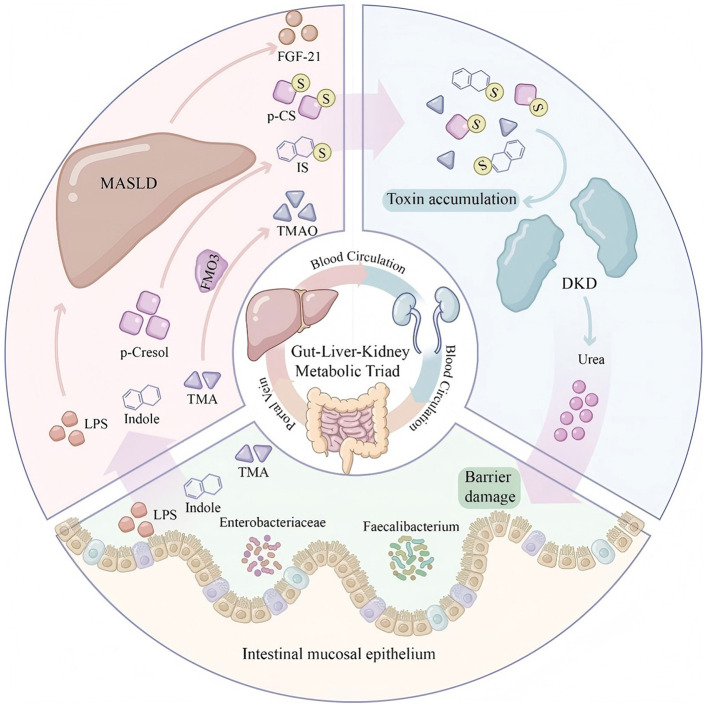
The gut-liver-kidney metabolic triad: inter-organ crosstalk and uremic toxin circulation. This triangular schematic illustrates the pathological inter-organ communication underlying the gut-liver-kidney axis in chronic kidney disease. **Intestine (bottom)**: dysbiotic microbiota metabolize dietary precursors to generate trimethylamine (TMA), indole, and p-cresol, while barrier dysfunction permits LPS translocation into portal circulation. **Liver (top left)**: hepatic flavin-containing monooxygenase 3 (FMO3) oxidizes TMA to trimethylamine N-oxide (TMAO). Indole and p-cresol undergo sulfation/glucuronidation to form indoxyl sulfate (IS) and p-cresyl sulfate (pCS), respectively. Progressive hepatic steatosis (MASLD) may exacerbate metabolic dysregulation. Hepatokines including FGF-21 enter systemic circulation. **Kidney (top right)**: declining glomerular filtration impairs clearance of TMAO, IS, and pCS, resulting in uremic toxin accumulation and promoting tubulointerstitial fibrosis characteristic of DKD. **The vicious cycle**: a prominent feedback arrow indicates urea transport from kidney to intestine, where urease-producing bacteria convert urea to ammonia, further compromising intestinal barrier integrity and perpetuating the cycle of metabolic toxicity. This circular causality underscores that the kidney functions not merely as a target organ but as an active participant in gut barrier deterioration. FMO3, flavin-containing monooxygenase 3; TMAO, trimethylamine N-oxide; IS, indoxyl sulfate; pCS, p-cresyl sulfate; FGF-21, fibroblast growth factor 21; DKD, diabetic kidney disease; MASLD, metabolic dysfunction-associated steatotic liver disease.

Alterations in tryptophan metabolic pathways are closely related to inflammatory responses in DKD. Gut microbiota excessively degrades tryptophan and phenylalanine, leading to increased nephrotoxic derivatives including phenyl sulfate and indole compounds (105). Kikuchi et al. (112) provided pioneering evidence that gut microbiome-derived phenyl sulfate contributes to albuminuria progression in DKD patients. These metabolites promote renal fibrosis through oxidative stress, mitochondrial dysfunction, and NLRP3 inflammasome activation (183). Lipid metabolic disorders also pervade the gut-liver-kidney axis, with DKD patients exhibiting widespread alterations across multiple lipid classes including triglycerides, cholesterol, sphingolipids, and phospholipids (184). The gut-liver-kidney axis establishes a pathological network in DKD, wherein uremic toxins (TMAO, IS, pCS) circulate through multi-organ coordination, and kidney-derived urea further damages intestinal barrier integrity, creating a vicious cycle of metabolic toxicity (Figure 5).

### 5.2 Intestinal barrier dysfunction and metabolic inflammation

#### 5.2.1 Association between intestinal leakage and DKD

Intestinal barrier dysfunction, termed “leaky gut,” has become a research hotspot in DKD pathogenesis. Compared with non-diabetic kidney disease, the gut leakage-renal injury cycle in DKD exhibits unique metabolic characteristics: chronic hyperglycemia selectively inhibits butyrate metabolic pathways, and the synergistic effects of advanced glycation end products (AGEs)-RAGE signaling with gut-derived toxins accelerate renal fibrosis (12, 185). Jiang et al. (185) systematically described how chronic hyperglycemia induces intestinal mucous layer depletion, distinguishing DKD from non-diabetic kidney disease.

Downregulation of intestinal epithelial tight junction proteins (ZO-1, occludin, and claudin-1) in DKD patients constitutes the molecular basis of gut leakage (183). This process is regulated by multiple factors: hyperglycemia directly induces intestinal mucous layer depletion; declining renal function leads to massive urea accumulation in the intestinal lumen, inducing tight junction protein downregulation (186); elevated TMAO levels can also downregulate tight junction proteins, establishing a destructive cycle (173). Linh et al. (187) revealed the critical role of mitochondrial antiviral signaling protein (MAVS) in maintaining intestinal integrity. MAVS-deficient diabetic mice exhibited more severe renal injury accompanied by gut leakage and Th17-mediated intestinal inflammation. Gut-derived *Klebsiella oxytoca* and elevated IL-17 activate the MAVS pathway in renal tubular epithelial cells, leading to kidney injury molecule-1 (KIM-1) production.

#### 5.2.2 Lipopolysaccharide-mediated systemic low-grade inflammation

Lipopolysaccharide (LPS), a surface antigen of Gram-negative bacteria, leaks into the portal venous circulation under gut leakage conditions and serves as a key molecule linking gut dysbiosis with systemic inflammation (6). LPS triggers endotoxemia and inflammatory responses through the Toll-like receptor (TLR)2/TLR4/NF-κB signaling pathway (183). Lin et al. (188) confirmed that TLR4-deficient diabetic mice exhibited reduced NF-κB activation, albuminuria, and renal dysfunction, validating the central role of the LPS-TLR4 axis in DKD inflammatory mechanisms.

The characteristic alterations in gut microbiota dysbiosis in DKD patients provide the foundation for LPS-mediated inflammation. Studies revealed that beneficial short-chain fatty acid (SCFA)-producing bacteria such as *Bifidobacterium* and *Lactobacillus* are decreased, while pathogenic bacteria such as Proteobacteria and *Bacteroides* are increased in DKD patients (169). This dysbiosis not only reduces SCFA production but also promotes endotoxin and pathogen translocation across the intestinal barrier, triggering inflammation and oxidative stress (189). Proteobacteria enrichment is an important feature of DKD gut microbiota, with *Escherichia-Shigella* enriched in feces of advanced CKD patients, contributing to renal function impairment through additional indoxyl sulfate production (190).

#### 5.2.3 Nutritional intervention strategies for restoring intestinal barrier

Short-chain fatty acids (SCFAs), particularly butyrate, show tremendous potential in restoring intestinal barrier function and improving DKD (191, 192). The clinical study by Zhong et al. (191) found that fecal acetate, propionate, and butyrate levels were significantly lower in DKD patients compared to normal controls (*p* < 0.001). Blood urea nitrogen was negatively correlated with fecal SCFAs, and urinary albumin-to-creatinine ratio (UACR) was negatively correlated with fecal acetate (*r* = −0.38, *p* < 0.01).

Huang et al. (193) systematically evaluated the effects of three major SCFAs in type 2 diabetic mouse models. The study found that exogenous SCFAs, particularly butyrate, improved hyperglycemia and insulin resistance, prevented proteinuria formation, and inhibited mesangial matrix accumulation and renal fibrosis. Mechanistic studies revealed that SCFAs suppress oxidative stress and NF-κB inflammatory signaling through the G protein-coupled receptor 43 (GPR43)-β-arrestin-2 pathway. Diep et al. (194) systematically summarized the protective effects of butyrate in various kidney injury models, with common mechanisms including anti-oxidative stress, anti-fibrosis, anti-inflammation, and anti-cell death effects.

The renoprotective effects of butyrate involve multiple molecular mechanisms. Du et al. (195) revealed that butyrate attenuates DKD through mediating the miR-7a-5p/P311/TGF-β1 pathway. Zhou et al. (196) found that exogenous sodium butyrate exerts anti-inflammatory and anti-fibrotic effects through inducing histone lysine butyrylation (Kbu), particularly H3K9 butyrylation. Dietary fiber supplementation shows potential for restoring intestinal barrier through enhancing the SCFAs-GPR43 signaling pathway (192). Polysaccharides such as *Bupleurum* polysaccharides exert renoprotective effects through modulating gut microbiota and inflammatory responses (197). Notably, Li et al. (198) found that isobutyrate was positively correlated with DN, and the additive interaction between high propionate and high isobutyrate significantly increased DN risk (OR) (193, 194), suggesting that SCFA effects are concentration-dependent and strain-specific.

### 5.3 Tripartite regulation of bile acid-microbiota-amino acid metabolism

#### 5.3.1 Effects of dietary components on bile acid profiles

Bile acids (BAs), serving as important signaling molecules, play critical regulatory roles in the gut-liver-kidney axis. Metabolomic studies revealed that bile acid metabolism exhibits stepwise changes during DKD progression (199, 200). Zhou et al. (199) employed ultra-high-performance liquid chromatography-tandem mass spectrometry to determine plasma BA profiles and found stepwise changes in BA metabolism during DKD progression. Alterations in bile acid profiles are closely related to glucolipid metabolic disorders in DKD patients, with elevated total bile acid (TBA) levels positively correlated with HOMA-IR index, and increased 12α-hydroxylated/non-12α-hydroxylated BA ratio accompanied by decreased insulin sensitivity (201).

Gut microbiota dysbiosis in DKD patients leads to decreased numbers of bacteria involved in bile acid synthesis and enterohepatic circulation, affecting various enzyme activities and reducing secondary bile acid synthesis. Decreased abundance of bile salt hydrolase (BSH)-producing *Bifidobacterium* and *Lactobacillus* leads to impaired Tβ-MCA deconjugation, enhancing FXR antagonism. Reduced 7α-dehydrogenase produced by *Bacteroides* in DKD patient intestines leads to decreased lithocholic acid (LCA) conversion, suppressing TGR5 excitatory activity (200). This microbiota-mediated bile acid profile remodeling provides new perspectives for understanding DKD metabolic disorders.

#### 5.3.2 Bile acid regulation of amino acid metabolic enzymes

Complex reciprocal regulatory relationships exist between bile acids and amino acid metabolism. Chen et al. (202) demonstrated that Tangshen Formula treatment remodels gut microbiota structure in aged DKD model rats and corrects tryptophan metabolism and arginine biosynthesis disorders. The study found that Tangshen Formula increased *Anaeroplasma* and *Barnesiella* genera, elevated tryptophan and 5-hydroxyindoleacetic acid levels, and reduced indole-3-acetic acid and xanthurenic acid levels. Correlation analysis indicated that gut microbiota dysbiosis was associated with tryptophan metabolism and arginine biosynthesis, jointly regulating inflammatory responses in aged DKD.

Bile acids can increase tissue glucose uptake and maintain systemic glucose homeostasis through activating FXR and TGR5 (203). This regulatory effect is partially achieved through influencing glucagon-like peptide-1 (GLP-1) secretion by intestinal L cells. Under normal conditions, SCFAs promote GLP-1 secretion through G protein-coupled receptors (GPR41/43) (33). In DKD, gut microbiota dysbiosis significantly reduces SCFA synthesis, particularly acetate and butyrate, leading to decreased GLP-1 secretion and exacerbating insulin resistance and renal injury (204). Lu et al. (204) revealed that intestinal dysbiosis activates the renin-angiotensin system, promoting incipient diabetic nephropathy development.

#### 5.3.3 FXR/TGR5 signaling and renal protection

The roles of bile acid receptors farnesoid X receptor (FXR) and Takeda G protein-coupled receptor 5 (TGR5) in DKD renal protection have received extensive attention in recent years (200, 205). FXR and TGR5 are widely expressed in the kidneys. Fang et al. (205) emphasized that dual receptor-deficient db/db mice exhibited elevated creatinine, uric acid, and proteinuria levels, suggesting that limited bile acid receptor expression may exacerbate renal injury in DKD patients.

Wang et al. (206) revealed different but complementary renoprotective pathways mediated by FXR and TGR5. TGR5 activation induces mitochondrial biogenesis, preventing renal oxidative stress and lipid accumulation. TGR5-deficient mice showed significantly reduced PGC-1α, ERRα, and SIRT3 protein expression in kidneys, while FXR-deficient mice were unaffected (207). Wang et al. (206) further found that the FXR/TGR5 dual agonist INT-767 demonstrated synergistic renoprotective effects in diabetic and obese mouse models, improving proteinuria, preventing podocyte injury, and attenuating tubulointerstitial fibrosis.

**Figure 6:**
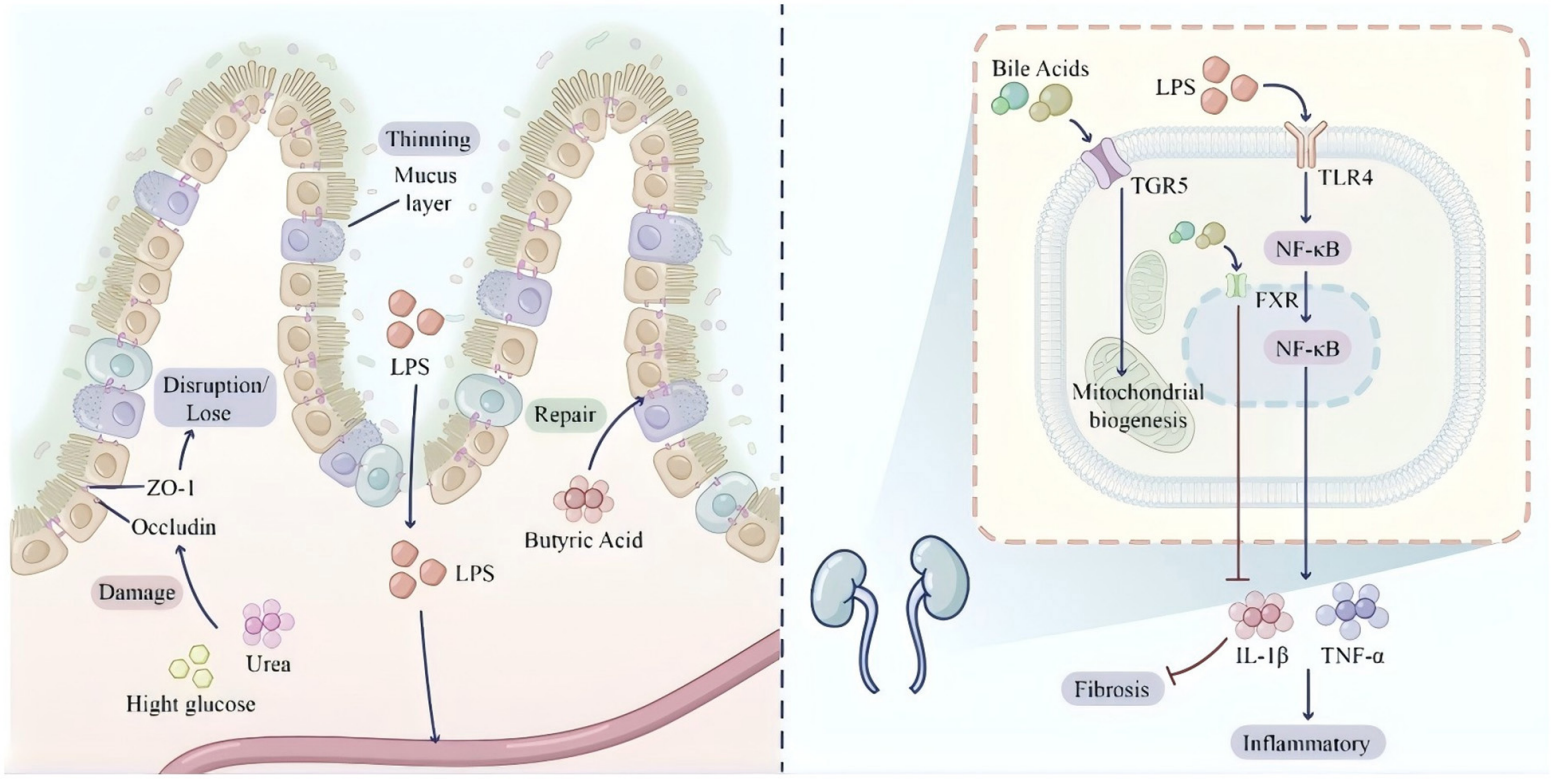
Intestinal barrier dysfunction and renal cellular signaling: mechanisms of leaky gut and downstream nephroprotective pathways. This split-screen mechanistic diagram details the molecular events connecting intestinal barrier failure to renal cellular responses. **Left panel (intestinal barrier dysfunction)**: the single-layer intestinal epithelium exhibits pathological features including disrupted tight junction proteins (ZO-1, occludin), attenuated mucus layer, and paracellular LPS translocation into subepithelial blood vessels. Hyperglycemia and elevated urea concentrations are depicted as barrier-damaging factors, while butyrate promotes tight junction restoration. **Right panel (renal proximal tubular cell signaling): damage pathway (red)**: LPS engagement of TLR4 activates NF-κB, which translocates to the nucleus and induces pro-inflammatory cytokine transcription (IL-1β, TNF-α). **Protection pathways**: bile acids, particularly dehydrolithocholic acid (DHLCA), activate membrane-bound TGR5 and nuclear FXR. TGR5 signaling enhances mitochondrial biogenesis (depicted as glowing mitochondria), while FXR activation suppresses transforming growth factor-β1 (TGF-β1) and attenuates fibrogenesis. This dual protective mechanism highlights potential therapeutic targets for preserving renal function.ZO-1, zonula occludens-1; LPS, lipopolysaccharide; TLR4, Toll-like receptor 4; NF-κB, nuclear factor kappa B; IL-1β, interleukin-1β; TNF-α, tumor necrosis factor-α; DHLCA, dehydrolithocholic acid; TGR5, Takeda G protein-coupled receptor 5; FXR, farnesoid X receptor; TGF-β1, transforming growth factor-β1.

Dehydrolithocholic acid (DHLCA), as a novel bile acid derivative, shows great potential in DKD treatment. Zhou et al. (199) found in DKD mouse models that DHLCA administration significantly reduced urinary albumin-to-creatinine ratio and fasting blood glucose levels (*p* < 0.01), improved liver function, attenuated renal tubular injury, and restored TGR5 and FXR expression in renal tissue. Metagenomic analysis revealed *Lachnospiraceae* enrichment following DHLCA treatment, suggesting its renoprotective effects are partially achieved through gut microbiota remodeling. DHLCA also exhibited hepatoprotective effects; this dual organ protection may be attributed to its dual regulation of metabolic and immune pathways. Various traditional Chinese medicine formulations, including Qidi Tangshen granules and Yi-Shen-Hua-Shi granules, have been confirmed to reconstruct intestinal microbial homeostasis while alleviating renal injury (208). The molecular mechanisms connecting intestinal barrier dysfunction to renal injury involve opposing signaling pathways: LPS-TLR4-NF-κB activation drives inflammation, while bile acid-mediated TGR5 and FXR signaling confer protection through enhanced mitochondrial biogenesis and suppressed fibrogenesis (Figure 6).

## 6 Nutritional intervention strategies targeting the gut microbiota-gut-liver-kidney axis with natural bioactive compounds

**Figure 7:**
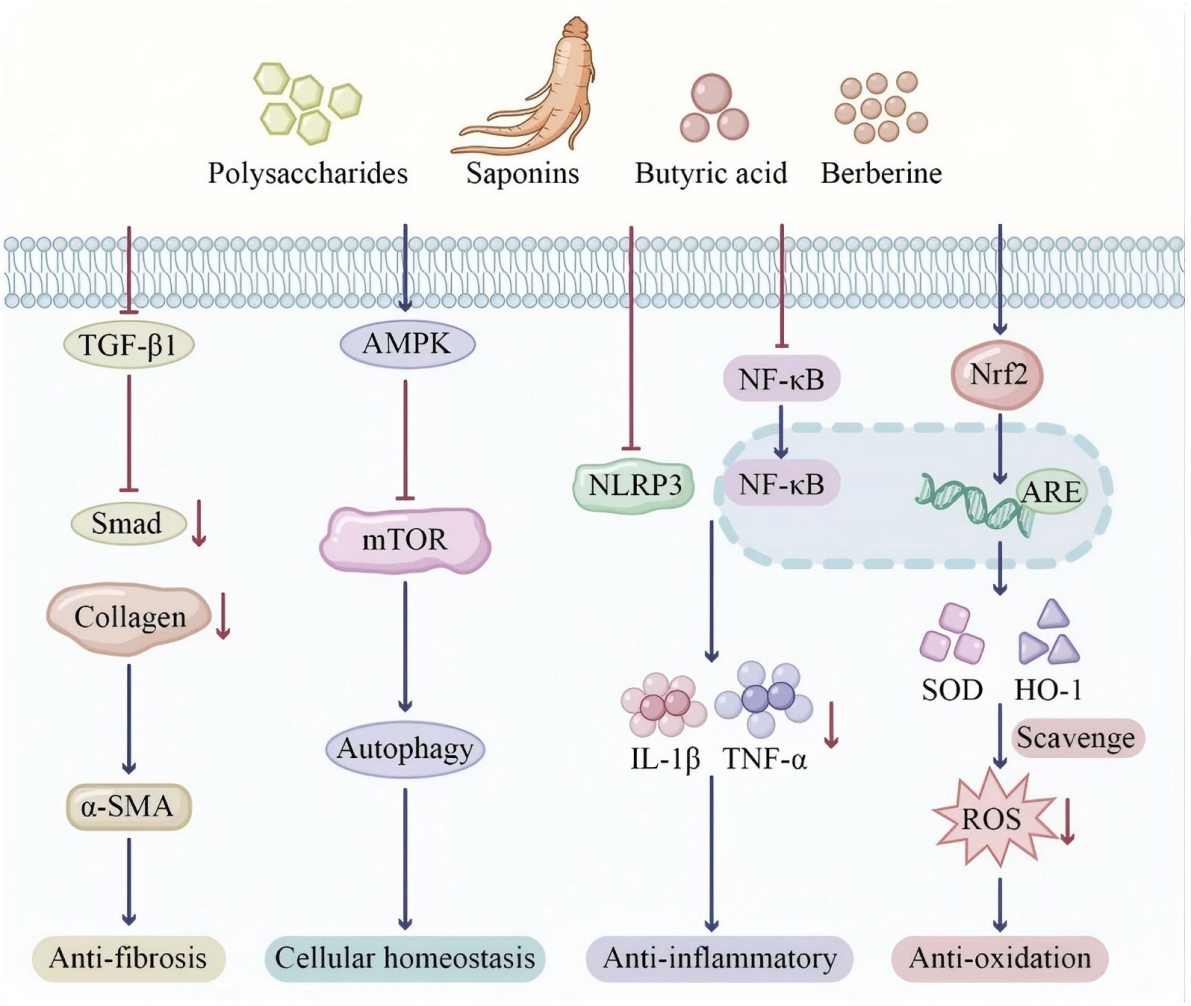
Triple-defense signaling map: natural compounds targeting inflammation, fibrosis, and oxidative stress in renal protection. This detailed cellular mechanism diagram depicts how bioactive natural compounds and their gut-derived metabolites simultaneously modulate three key pathological pathways in renal tubular cells and podocytes. **Extracellular inputs**: natural compounds including polysaccharides (GLP), curcumin, berberine, SCFAs (butyrate), and ginsenosides enter renal cells or engage membrane receptors. **Intracellular triple defense mechanisms: anti-fibrosis (left)**: inhibition of TGF-β1/Smad signaling reduces collagen deposition and α-smooth muscle actin (α-SMA) expression, attenuating renal scarring. **Anti-inflammation (center)**: blockade of NF-κB nuclear translocation and NLRP3 inflammasome assembly diminishes IL-1β and TNF-α production. **Anti-oxidation (right)**: Nrf2 nuclear translocation and antioxidant response element (ARE) binding upregulate heme oxygenase-1 (HO-1) and superoxide dismutase (SOD), enhancing reactive oxygen species (ROS) scavenging capacity. **Central regulatory hub**: AMP-activated protein kinase (AMPK) activation suppresses mTOR signaling, thereby inducing autophagy and promoting cellular quality control. **Visual annotation (top left corner)**: a small intestine icon with arrow indicates that these protective compounds originate from gut microbial metabolism, reinforcing the gut-kidney axis concept. T-bars represent inhibition; arrows indicate activation. TGF-β1, transforming growth factor-β1; α-SMA, α-smooth muscle actin; NF-κB, nuclear factor kappa B; NLRP3, NOD-like receptor protein 3; IL-1β, interleukin-1β; TNF-α, tumor necrosis factor-α; Nrf2, nuclear factor erythroid 2-related factor 2; ARE, antioxidant response element; HO-1, heme oxygenase-1; SOD, superoxide dismutase; ROS, reactive oxygen species; AMPK, AMP-activated protein kinase; mTOR, mechanistic target of rapamycin.

Natural bioactive compounds exert renoprotection through a triple-defense mechanism: suppressing TGF-β1/Smad-mediated fibrosis, inhibiting NF-κB/NLRP3-driven inflammation, and activating Nrf2/ARE-dependent antioxidant responses, with AMPK serving as a central metabolic hub (Figure 7). Where Table 2 summarizes the clinical application parameters of natural bioactive compounds in the treatment of diabetic kidney disease.

**Table 2:** Clinical application parameters of natural bioactive compounds in diabetic kidney disease.

Compound class	Representative agent	Effective dose range (human studies)	Oral bioavailability (%)	Primary safety concerns	DKD-specific considerations	Key references
Flavonoids	Quercetin	500–1,000 mg/day	< 5%	High doses may affect CYP3A4; potential interaction with anticoagulants	Risk of metabolite accumulation with declining eGFR; nanoformulations recommended	(211, 212, 253)
	Baicalin	400–800 mg/day	2.2%	Generally well-tolerated; mild gastrointestinal discomfort at high doses	Modulates bile acid metabolism; monitor with RAAS inhibitors	(214)
	Rutin	500–1,500 mg/day	15%−20% (after microbial conversion)	Low toxicity; requires gut microbiota α-rhamnosidase	Individual variability due to microbiota composition	(215, 314)
Phenolic acids	Chlorogenic acid	140–420 mg/day	33%	Generally safe; high doses may cause gastric irritation	Protects intestinal barrier; synergistic with GLP-1 pathway	(217, 218, 315)
	Ferulic acid	50–500 mg/day	42%−52%	Minimal adverse effects	Promotes *Akkermansia*; monitor potassium with RAAS inhibitors	(219, 316)
Polysaccharides	Astragalus polysaccharides	7.5–15 g/day	N/A (colonic fermentation)	Caution in autoimmune conditions	SCFA production depends on baseline microbiota	(221, 269–271)
	Inulin	10–20 g/day	N/A (colonic fermentation)	Initial bloating; gradual escalation recommended	Reduces uremic toxins; avoid in CKD stage 5	(150, 317)
	Resistant starch (RS2)	15–40 g/day	N/A (colonic fermentation)	Monitor for hyperkalemia in CKD stages 4–5	Modulates microbiota; individualize dose by eGFR	(151, 318)
Alkaloids	Berberine	500 mg 2–3 times/day	< 1%	Gastrointestinal discomfort; avoid in pregnancy	Activates hepatic FXR; dose adjustment at eGFR < 30	(227, 228)
Saponins	Ginsenoside Rb1	10–20 mg/day	0.1% (requires microbial conversion to compound K: ~14%)	Caution with anticoagulants and antidiabetic drugs	Biotransformation depends on *Bacteroides* abundance	(230, 231, 319)
Terpenoids	Curcumin	500–2,000 mg/day	< 1%	High doses may cause nausea; avoid in bile duct obstruction	Dose reduction at eGFR < 45; lipid formulations improve bioavailability 29-fold	(232, 233, 255)
	Allicin	600–1,200 mg aged garlic extract/day	Variable (S-allyl-cysteine: ~98%)	Breath/body odor; potentiates anticoagulants	Modulates Nrf2/HO-1 pathway; monitor glucose with sulfonylureas	(234, 235, 320)

### 6.1 Plant polyphenolic compounds

Plant polyphenols are secondary metabolites widely present in plant-based foods, possessing significant antioxidant, anti-inflammatory, and microbiota-modulating properties (209). Polyphenolic compounds undergo microbial metabolic transformation in the gut to produce biologically active metabolites, while simultaneously regulating gut microbiota composition, thereby establishing a bidirectional polyphenol-microbiota interaction network (210).

#### 6.1.1 Flavonoid compounds

Flavonoid compounds represent the most extensively studied subclass of the polyphenol family. Quercetin, as a representative flavonol, has demonstrated significant renoprotective effects in CKD models. Peng et al. (211) demonstrated in a murine model that quercetin ameliorated hyperuricemic nephropathy by improving gut dysfunctions and decreasing gut bacteria-derived uremic toxins [Animal model evidence]. Multi-omics analysis revealed that high-dose quercetin downregulated Blautia, suppressed microbial phenylalanine metabolism pathways, and significantly reduced nephrotoxic metabolites. However, clinical translation of these findings requires verification in human trials, as interspecies differences in gut microbiota composition and xenobiotic metabolism may affect therapeutic efficacy.

Multi-omics analysis revealed that high-dose quercetin downregulated *Blautia*, a key gut bacterium associated with uremic toxin production, suppressed microbial phenylalanine metabolism pathways, and significantly reduced nephrotoxic metabolites including 3-phenyllactic acid, hippuric acid, and N-acetyl-L-phenylalanine.

Mi et al. (212) demonstrated that quercetin treatment selectively enriches Verrucomicrobia and Akkermansia genera, positively affecting gene expression profiles and metabolic pathways of gut microbiota. Such microbiota remodeling may contribute to improved metabolic homeostasis and reduced production of harmful metabolites. Studies have shown that phenolic acid compounds such as protocatechuic acid can ameliorate inflammatory responses by modulating gut microbiota and upregulating intestinal tight junction protein expression (213). Quercetin, a representative flavonol compound, demonstrates significant renoprotective effects in CKD models. A recent study showed quercetin ameliorates hyperuricemic nephropathy by improving gut dysfunctions and decreasing gut bacteria-derived uremic toxins (211). Multi-omics analysis revealed high-dose quercetin downregulated *Blautia*, a key gut bacterium associated with uremic toxin production, suppressed microbial phenylalanine metabolism pathways, and significantly reduced nephrotoxic metabolites including 3-phenyllactic acid, hippuric acid, and N-acetyl-L-phenylalanine (211).

Baicalin, a flavonoid glycoside extracted from *Scutellaria baicalensis*, possesses unique microbiota-modulating properties. This compound exerts hepatoprotective effects through promoting short-chain fatty acid-producing bacteria proliferation, regulating TGR5 and FXR signaling pathways, and modulating bile acid metabolism (214). The microbiota-modulating mechanism may confer similar protective effects in CKD-related hepatorenal injury.

Rutin, as a glycosidic form of quercetin, depends on gut microbial α-rhamnosidase activity for its bioavailability. Gut microbiota metabolizes rutin into quercetin and other metabolites that enter the liver via the portal vein to exert biological effects (215). The interaction between flavonoid compounds and gut microbiota determines their bioavailability.

The presence of gut microbiota can significantly enhance plasma concentrations and bioavailability of quercetin (210). Quercetin promotes the proliferation of short-chain fatty acid (SCFA)-producing bacteria such as *Faecalibacterium prausnitzii* and *Roseburia*, enhancing intestinal barrier function through butyrate production via dietary fiber fermentation. Anthocyanin polyphenols can modulate gut microbiota composition and exhibit anti-inflammatory and intestinal protective effects (216).

#### 6.1.2 Phenolic acid compounds

Phenolic acid compounds primarily include hydroxycinnamic acids (such as chlorogenic acid, ferulic acid, and caffeic acid) and hydroxybenzoic acids, which are abundant in coffee, tea, and whole grains. Chlorogenic acid (CGA) is the major polyphenol component in coffee. Shi et al. (217) found that CGA can ameliorate high-fat diet-induced non-alcoholic fatty liver disease, increasing *Bifidobacterium* content, reducing *Escherichia coli* content, upregulating intestinal tight junction proteins ZO-1 and Occludin expression, decreasing portal vein LPS levels, and elevating GLP-1 levels. Li et al. (218) further confirmed that CGA combined with geniposide improves non-alcoholic steatohepatitis through the FXR signaling pathway by regulating gut microbiota metabolism and bile acid profiles.

Ferulic acid is abundant in whole grains and traditional Chinese medicines such as *Angelica sinensis* and *Ligusticum chuanxiong*. Studies have shown that ferulic acid intervention can increase the relative abundance of *Akkermansia muciniphila*, promote intestinal mucus layer thickening, and reduce intestinal permeability. Caffeic acid can exert protective effects in colitis models by increasing the abundance of *Akkermansia* in the gut (219), and this mechanism may also apply to CKD-related intestinal inflammation.

### 6.2 Dietary polysaccharides and prebiotics

#### 6.2.1 Prebiotic effects of plant polysaccharides

Dietary polysaccharides are carbohydrates not degraded by human digestive enzymes and are fermented by gut microbiota in the colon to produce beneficial metabolites such as SCFAs. According to the International Scientific Association for Probiotics and Prebiotics consensus definition, prebiotics are substrates selectively utilized by host microorganisms to confer health benefits (220).

Traditional Chinese medicine polysaccharides possess significant immunomodulatory and organ-protective effects. Astragalus polysaccharides (APS) are water-soluble polysaccharides extracted from *Astragalus membranaceus* roots. Hong et al. (221) confirmed through integrated metagenomic and metabolomic analyses in mice that APS intervention can significantly ameliorate high-fat diet-induced metabolic disorders [Animal model evidence], altering the abundance of 188 species and reversing changes in 36 metabolites. Glutathione metabolism and purine metabolism pathways were identified as key regulatory pathways. While these preclinical findings are promising, the dose-response relationship and metabolic effects in humans remain to be established through randomized controlled trials.

Rong et al. (222) found through metatranscriptomic analysis that the synergistic action of Astragalus polysaccharides and *Codonopsis pilosula* polysaccharides can significantly upregulate key genes involved in butyrate synthesis in gut microbiota, including butyryl-CoA:acetate-CoA transferase and butyrate kinase. In addition to Astragalus polysaccharides, other traditional Chinese medicine-derived polysaccharides also demonstrate significant prebiotic effects.

*Lycium barbarum* polysaccharides (LBP) are bioactive polysaccharides extracted from goji berries. LBP significantly ameliorated DSS-induced chronic ulcerative colitis, increasing the abundance of *Lactobacillus, Butyricicoccus*, and *Akkermansia*, promoting tight junction protein ZO-1 and Occludin expression, and elevating fecal short-chain fatty acid levels (223).

*Ganoderma lucidum* polysaccharides (GLP) possess β-glucan structural characteristics. GLP inhibits hepatic stellate cell activation and liver fibrosis by targeting inflammation, apoptosis, and cell cycle through the TGF-β/Smad signaling pathway (224). GLP can also improve liver function by regulating gut microbiota, significantly increasing fecal SCFA content. Butyrate upregulates intestinal epithelial tight junction protein expression by activating GPR109A/GPR43 receptors, improving intestinal permeability.

#### 6.2.2 Functional dietary fiber

Functional dietary fibers are fermentable carbohydrates with specific health benefits. Inulin is a fructan extracted from chicory root. Esgalhado et al. (150) systematically reviewed the role of SCFAs as a link between prebiotics and microbiota in CKD. Inulin supplementation can reduce serum uremic toxin levels, increase *Bifidobacterium* relative abundance, and reduce urease-producing *Klebsiella* and *Proteus*.

Fructooligosaccharides (FOS) are short-chain forms of inulin. Rossi et al. (225) conducted the SYNERGY randomized controlled trial comparing the effects of synbiotics on hemodialysis patients. Serum indoxyl sulfate and p-cresyl sulfate decreased significantly after intervention, with good tolerability. Synbiotic intervention significantly reduced serum inflammatory markers IL-6 and TNF-α.

Resistant starch (RS) is starch that is not digested and absorbed in the small intestine. Headley et al. (151) conducted a randomized controlled trial evaluating the efficacy of high-amylose maize starch (RS2) in CKD stage 3a-4 patients. Daily RS2 supplementation can modulate gut microbiota composition, reduce uremic toxin levels, and improve renal function indicators. The presence of fermentable carbohydrates causes gut microbiota to preferentially utilize carbohydrates as an energy source, reducing protein fermentation (100).

### 6.3 Alkaloid bioactive compounds

Alkaloids are nitrogen-containing basic organic compounds widely present in various traditional Chinese medicines. Berberine (also known as huang lian su) is an isoquinoline alkaloid extracted from *Coptis chinensis* and *Phellodendron amurense*, possessing multi-target metabolic regulatory effects (226).

Habtemariam (227) systematically reviewed the gut microbiota-modulating effects of berberine and its applications in metabolic diseases. In CKD models, berberine intervention can significantly improve renal function and regulate gut microbiota composition. 16S rRNA sequencing showed significantly increased relative abundance of *Akkermansia muciniphila* and *Lactobacillus*.

The relationship between berberine's regulation of microbiota composition and bile acid metabolism is particularly important. Zhang et al. (228) revealed the gut microbiome-related effects of berberine and probiotics on type 2 diabetes through the PREMOTE study. Berberine intervention significantly altered bile acid profiles in the gut and serum, with increased fecal excretion of primary bile acids and decreased secondary bile acids. Mechanistic studies showed that berberine inhibited the growth of bacteria involved in bile acid 7α-dehydroxylation, and changes in bile acid composition activated hepatic FXR signaling pathway.

### 6.4 Saponin compounds

Saponins are compounds formed by the combination of triterpene or steroid aglycones with sugar chains, abundantly present in traditional Chinese medicines such as ginseng, Astragalus, and *Panax notoginseng*. These compounds exert comprehensive regulation of the liver-kidney axis by improving insulin sensitivity and renal function.

Ginsenosides are the main active components of ginseng. Qi et al. (229) systematically reviewed the chemical diversity and pharmacological activities of ginsenosides. Zou et al. (230) evaluated the efficacy of ginsenoside Rb1 in high-fat diet-induced obese mice, finding that Rb1 improved glucose and lipid metabolism disorders closely related to gut microbiota modulation. 16S rRNA sequencing showed that Rb1 intervention increased the abundance of SCFA-producing bacteria. Zhou et al. (231) reviewed that ginsenoside Rb1 can improve oral bioavailability, modulate gut microbiota composition/balance, increase gut permeability, and stimulate GLP-1 secretion to exert hypoglycemic and anti-diabetic effects, and can ameliorate diabetic complications including diabetic nephropathy.

The biotransformation of saponin compounds is dependent on gut microbiota. Specifically, Bacteroides species possess strong glycosidase activity, enabling the progressive hydrolysis of ginsenoside Rb1 into metabolites such as compound K, which typically exhibit higher biological activity than their parent compounds (229).

### 6.5 Terpenoids and volatile components

Terpenoids are natural products polymerized from isoprene units, widely distributed in plants and serving important physiological functions. Volatile components are mostly monoterpenes and sesquiterpenes, possessing significant anti-inflammatory and metabolic regulatory activities.

Curcumin is a diarylheptanoid compound extracted from turmeric rhizomes. Khajehdehi et al. (232) conducted a randomized controlled trial evaluating the renoprotective effects of curcumin in type 2 diabetic nephropathy patients. Patients were randomly assigned to receive curcumin or placebo treatment. Results showed that the curcumin group had significantly reduced proteinuria and decreased serum TGF-β and IL-8 levels. Shen et al. (233) found that curcumin can significantly alter gut microbiota composition, with increased relative abundance of *Akkermansia muciniphila*, and functional prediction analysis showed that curcumin enriched genes involved in bile acid metabolism, activating hepatic FXR and TGR5 signaling pathways.

**Figure 8:**
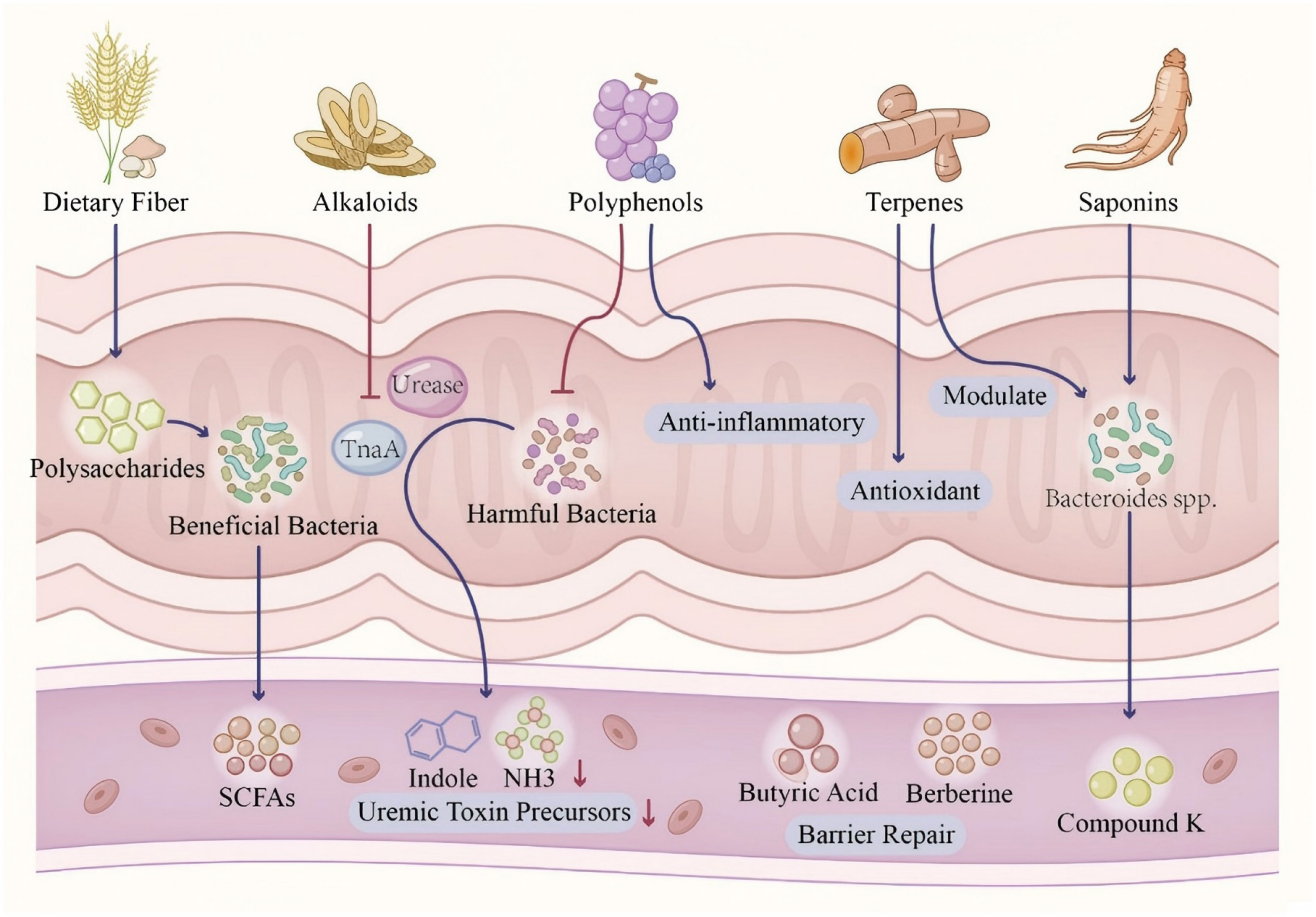
Natural dietary compounds as modulators of gut microbiota and metabolic homeostasis. This comprehensive schematic illustrates how five major classes of natural dietary compounds reshape the gut microbial ecosystem and metabolic output. **Dietary inputs (top)**: five distinct compound classes are depicted with representative icons: (1) Polysaccharides (grains/mushrooms); (2) Alkaloids (*Coptis chinensis*/berberine); (3) Polyphenols (grapes/berries); (4) Terpenes (garlic/*Curcuma*); and (5) Saponins (ginseng root). **Intestinal biochemical processes (middle): prebiotic effect**: polysaccharides selectively nourish beneficial bacteria (*Akkermansia, Bifidobacterium*), enhancing SCFA production (green glowing particles). **Enzyme inhibition**: anthocyanins and alkaloids suppress pathogenic bacteria (*Proteus, Escherichia coli*) by inhibiting tryptophanase (TnaA) and urease, thereby reducing indole and ammonia generation. **Biotransformation**: ginsenoside Rb1 undergoes bacterial conversion by *Bacteroides* to bioactive compound K. **Functional outcomes (bottom)**: berberine and butyrate cooperatively restore tight junction integrity (zipper-like structure). Blue arrows indicate beneficial metabolite flux into circulation, while blocked red arrows signify reduced uremic toxin production. The transition from dysbiosis to eubiosis is represented by split background coloring. SCFAs, short-chain fatty acids; TnaA, tryptophanase.

Allicin is the main volatile sulfur-containing compound in garlic. Zhai et al. (234) found that S-allyl-cysteine sulfoxide (alliin) has significant hypoglycemic and hypolipidemic effects in diet-induced obese mouse models, and these metabolic regulatory effects may help alleviate CKD-related metabolic disturbances. Allicin and its metabolites exert immunomodulatory and anti-inflammatory effects by activating the Nrf2/HO-1 pathway and inhibiting NF-κB/NLRP3 inflammasome (235). Natural bioactive compounds from five major classes—polyphenols, polysaccharides, alkaloids, saponins, and terpenes—exert gut-protective effects through distinct but complementary mechanisms: prebiotic enrichment of beneficial bacteria, enzymatic inhibition of uremic toxin production, and enhancement of intestinal barrier integrity (Figure 8).

### 6.6 Bioavailability enhancement and metabolite attribution

#### 6.6.1 Bioavailability challenges of polyphenols and polysaccharides

Clinical translation of natural bioactive compounds confronts a critical bottleneck: remarkably low oral bioavailability. The majority of polyphenolic compounds and polysaccharides exhibit bioavailability below 10%, primarily attributable to poor aqueous solubility, chemical instability under gastrointestinal pH conditions, extensive hepatic first-pass metabolism, and limited membrane permeability (236, 237). Quercetin demonstrates oral bioavailability ranging from 2 to 17% (238), while curcumin exhibits bioavailability below 1% due to rapid metabolic degradation and inadequate absorption (239). Polysaccharides, though possessing greater stability, face absorption barriers related to their high molecular weight (typically >10 kDa), which restricts both paracellular and transcellular intestinal transport (240).

Following oral administration, polyphenols undergo extensive phase II metabolism (glucuronidation, sulfation, and methylation), generating metabolites with altered biological activities (241). This metabolic transformation raises a fundamental question: do the observed therapeutic effects originate from parent compounds or their derivatives?

#### 6.6.2 Gut microbiota-mediated biotransformation and mechanistic attribution

##### 6.6.2.1 Microbial metabolic transformation pathways

The gut microbiota functions as a “metabolic organ,” extensively biotransforming natural compounds into secondary metabolites with enhanced bioavailability and biological activity (242). Microbial metabolism proceeds through a biphasic process: bacterial glycosidases initially catalyze deglycosylation to liberate aglycones, followed by ring-fission and reduction reactions generating diverse phenolic acids and derivatives (243).

Intestinal bacteria *Eubacterium ramulus* and *Flavonifractor plautii* cleave the quercetin C-ring, producing 3,4-dihydroxyphenylacetic acid and other metabolites that circulate at substantially higher concentrations than the parent compound (244). Anthocyanins undergo rapid degradation by colonic microbiota into protocatechuic acid and gallic acid, exhibiting superior bioavailability and vascular protective effects (245). Ginsenoside Rb1 experiences sequential deglycosylation by *Bacteroides* species, ultimately yielding compound K (CK) with 15-fold higher bioavailability than Rb1; the negligible therapeutic response of germ-free mice to Rb1 supplementation confirms the essential role of microbial transformation (246, 247).

##### 6.6.2.2 Synergistic attribution of the “triple-defense” mechanism

The “triple-defense” mechanism illustrated in Figure 7 represents synergistic actions of both parent compounds and gut microbiota-derived metabolites, rather than singular attribution. Intact polyphenols/polysaccharides that escape microbial degradation in the upper gastrointestinal tract directly activate signaling pathways in intestinal epithelial cells and immune cells (233). Following absorption, microbiota-derived metabolites enter systemic circulation and accumulate in renal tissue to exert protective effects. Urolithin A, an ellagitannin metabolite, activates mitophagy and improves mitochondrial function in renal tubular cells (248). Equol, derived from daidzein, exerts anti-inflammatory effects through estrogen receptor β activation (249). The changes in glycated hemoglobin, as the primary outcome, in the probiotics+BBR [least-squares mean (95% CI), −1.04 (−1.19, −0.89)%] and BBR-alone group [−0.99 (−1.16, −0.83)%] were significantly greater than that in the placebo and probiotics-alone groups [−0.59 (−0.75, −0.44)%, −0.53 (−0.68, −0.37)%, *p* < 0.001]. Further metagenomics and metabolomic studies found that the hypoglycaemic effect of BBR is mediated by the inhibition of DCA biotransformation by Ruminococcus bromii (228).

Therapeutic efficacy emerges from integrated actions along the gut-liver-kidney axis: (1) local intestinal effects of parent compounds on barrier integrity and immune modulation; (2) hepatic processing of absorbed aglycones; (3) systemic distribution of microbiota-derived phenolic acids; and (4) renal target engagement by circulating metabolites.

Table 3 summarizes the gut microbiota-mediated transformation of natural products and the subsequent renoprotective mechanisms.

**Table 3:** Gut microbiota-mediated transformation of major natural products and renoprotective mechanisms.

Parent compound	Metabolizing bacteria	Primary metabolites	Bioavailability enhancement	Renoprotective mechanisms	References
Quercetin	*Eubacterium* spp.	3,4-Dihydroxyphenylacetic acid	5–10 fold	Nrf2 activation, NF-κB inhibition	(244)
Anthocyanins	*Bifidobacterium* spp.	Protocatechuic acid, Gallic acid	8–15 fold	Endothelial protection, Antioxidation	(245)
Ginsenoside Rb1	*Bacteroides fragilis*	Compound K	15–20 fold	mTOR inhibition, Autophagy induction	(246, 247)
Ellagitannins	Urolithin producers	Urolithin A	20–40 fold	Mitophagy activation	(248)
Daidzein	Equol producers	Equol	10–30 fold	ERβ activation, Anti-inflammation	(249)
Berberine	*Eggerthella* spp.	Dihydroberberine	5–8 fold	Bile acid modulation, FXR activation	(228)

#### 6.6.3 Advances and limitations of nanocarrier technologies

##### 6.6.3.1 Biopolymer-based nanodelivery systems

Biopolymer-based nanocarriers (protein nanoparticles, polysaccharide nanogels, lipid nanostructures) offer superior advantages over synthetic polymers through biocompatibility, biodegradability, and Generally Recognized as Safe (GRAS) status (250, 251). Chitosan nanoparticles (50–200 nm) enhance quercetin bioavailability 5.8-fold through mucoadhesive properties that prolong intestinal residence time. Zein-pectin core-shell nanoparticles protect curcumin from gastric degradation while achieving 89.4% encapsulation efficiency and 6.2-fold bioavailability enhancement (252). Polysaccharide-based nanogels demonstrate dual functionality: serving as delivery vehicles while independently modulating gut microbiota composition. Human pharmacokinetic studies demonstrated that curcumin-phospholipid nanoparticles increased area under the curve (AUC) by 29-fold and maximum plasma concentration (Cmax) by 65-fold (250).

##### 6.6.3.2 Economic feasibility and alternative strategies

**Cost barriers**: pharmaceutical-grade nanoformulation manufacturing costs range from $50–500 per gram of active ingredient, representing a 10–100 fold increase over conventional extracts. For DKD patients requiring decades-long therapy, a 500mg daily polyphenol dose translates to $25–250 per day. Health economic modeling reveals that the 10-year incremental cost-effectiveness ratio (ICER) exceeds $500,000 per quality-adjusted life year (QALY), far above acceptable thresholds ($50,000–100,000/QALY), rendering this approach economically unsustainable.

**Gut microbiota-leveraging strategies**: synbiotic combinations (prebiotics + probiotics) offer superior cost-effectiveness. A 12-week clinical trial in CKD patients demonstrated that inulin plus *Lactobacillus plantarum* co-administered with cranberry extract increased circulating microbiota-derived phenolic acids by 340%, achieving bioavailability comparable to nanoformulations at 1/50th the cost (251). Personalized microbiome profiling ($100–200) followed by targeted dietary interventions represents a one-time investment. Individuals with high abundance of urolithin-producing bacteria derive significantly greater cardiovascular benefits from pomegranate consumption (252).

**Whole food matrix approach**: clinical evidence demonstrates that daily consumption of 50 g mixed berries (providing ~500 mg anthocyanins) improved glycemic control and reduced albuminuria in diabetic patients comparably to purified extracts, at < $1/day (250). Hemodialysis patients consuming 250 mL/day pomegranate juice (providing ellagitannins) achieved oxidative stress marker reduction matching high-dose supplements (251).

### 6.7 Bioavailability enhancement strategies and DKD application challenges

Clinical translation of natural bioactive compounds in DKD management is significantly limited by poor oral bioavailability, rapid metabolism, and renal clearance alterations. This section systematically addresses pharmaceutical strategies to overcome these barriers and discusses DKD-specific pharmacokinetic considerations.

#### 6.7.1 Mechanisms underlying poor bioavailability

Most polyphenols, flavonoids, and terpenoids exhibit oral bioavailability < 10% due to poor aqueous solubility, extensive Phase II metabolism (glucuronidation, sulfation) in enterocytes and hepatocytes, P-glycoprotein-mediated intestinal efflux, and rapid biliary and renal excretion of conjugated metabolites (236). Quercetin's bioavailability is < 5% because 95% undergoes first-pass metabolism to form quercetin-3-glucuronide and quercetin3′-sulfate, which are rapidly excreted via renal organic anion transporters (OAT1/3) (253).

#### 6.7.2 Pharmaceutical formulation strategies

**A. Nanoparticle delivery systems**

Encapsulation of bioactive compounds in nanoparticles (10–200 nm) enhances bioavailability by increasing surface area and dissolution rate, protecting against gastric degradation, facilitating lymphatic absorption to bypass first-pass metabolism, and achieving sustained release (254) phospholipid formulation improved curcumin bioavailability 29-fold in humans, with AUC increasing from 0.51 μg·h/mL (free curcumin 2 g) to 14.9 μg·h/mL (SLN-curcumin 410 mg) (255).

**B. Phospholipid complexation (phytosomes)**

Phytosomes are molecular complexes formed between polyphenols and phosphatidylcholine, enhancing lipophilicity and membrane permeability. Phospholipid complexation facilitates passive diffusion across enterocytes and protects against Phase II enzymes (256).

#### 6.7.3 DKD-specific pharmacokinetic alterations

**A. Impact of declining renal function**

Reduced eGFR profoundly affects the pharmacokinetics of renally cleared metabolites. Quercetin-3-glucuronide clearance decreases from 420 mL/min (eGFR >90) to 95 mL/min (eGFR 30–44), resulting in 4.4-fold higher plasma concentrations (253). Although glucuronide conjugates have lower biological activity, excessive accumulation may cause unpredictable effects or compete with endogenous substrate metabolism (257).

CKD-associated gut microbiota dysbiosis impairs the deconjugation and 7α-dehydroxylation of bile acids, reducing the formation of secondary bile acids that activate TGR5 (200). This diminishes the renoprotective effects of polysaccharides and saponins that rely on bile acid signaling (199).

**Dose adjustment recommendations:**

- **eGFR 45–59 mL/min/1.73 m**^2^ **(CKD stage 3a)**: no adjustment for most compounds; monitor hepatic function
- **eGFR 30–44 mL/min/1.73 m**^2^ **(CKD stage 3b)**: reduce quercetin, curcumin, and berberine doses by 25%−50%; use enhanced-bioavailability formulations
- **eGFR 15–29 mL/min/1.73 m**^2^ **(CKD stage 4)**: reduce doses by 50%; avoid high-dose polyphenols (>500 mg/day)
- **eGFR** **< 15 mL/min/1.73 m**^2^ **(CKD stage 5)**: use with extreme caution; nanoformulations preferred; monitor for adverse effects.

**B. Drug-nutrient interactions in DKD**

APS-derived SCFAs activate renal tubular AMPK and GPR43, potentially synergizing with SGLT2 inhibitors to enhance natriuresis and reduce tubular injury (20, 193). However, combined therapy may increase urinary glucose excretion, theoretically promoting Candida overgrowth—clinical vigilance required.

Ferulic acid and APS promote *Akkermansia muciniphila*, which produces propionate that inhibits renal angiotensinogen expression via GPR43 activation (258). This may enhance the antihypertensive and antiproteinuric effects of ACE inhibitors and ARBs. Conversely, berberine's potent CYP3A4 inhibition may increase plasma levels of valsartan by 40%, necessitating dose titration (259).

Berberine shares overlapping mechanisms with metformin (AMPK activation, improved insulin sensitivity), and combined use may increase hypoglycemia risk. A meta-analysis reported 0.71% greater HbA1c reduction with berberine plus metformin vs. metformin alone, but also 2.3-fold higher incidence of gastrointestinal side effects (260).

#### 6.7.4 Precision dosing via pharmacokinetic modeling

Emerging physiologically-based pharmacokinetic (PBPK) models integrate patient-specific factors (age, body weight, eGFR, CYP450 genotype, microbiota composition) to predict optimal dosing regimens (261).

**Future directions:**

- **Microbiota-informed dosing**: quantifying *Bacteroides* abundance to predict ginsenoside Rb1 biotransformation into compound K, enabling personalized saponin dosing (262).
- **Therapeutic drug monitoring**: developing point-of-care assays to measure plasma polyphenol metabolites, guiding dose adjustments in real-time.
- **AI-driven pharmacokinetics**: machine learning algorithms trained on multi-omics data to optimize compound combinations and predict individual responses (263).

#### 6.7.5 Overcoming renal targeting challenges

While systemic bioavailability is crucial, renal-specific delivery remains a major challenge. Nanocarriers functionalized with kidney-targeting ligands (megalin-binding peptides, low-molecular-weight chitosan) achieve 3–5-fold higher renal accumulation compared to non-targeted formulations (264). A proof-of-concept study demonstrated curcumin-loaded, megalin-targeted liposomes reduced diabetic glomerular hypertrophy by 58% at one-fifth the dose of free curcumin (265).

**Conclusion:** pharmaceutical innovations—nanoparticles, phytosomes, bioenhancers, and targeted delivery—have the potential to unlock clinical efficacy of natural bioactive compounds in DKD. However, DKD-specific pharmacokinetic alterations necessitate individualized dose adjustments based on eGFR, concomitant medications, and gut microbiota status. Large-scale clinical trials incorporating these enhanced formulations are urgently needed to establish evidence-based dosing guidelines for DKD patients across all CKD stages.

## 7 Development and application of medicine food homology substances and functional foods

### 7.1 Medicine food homology concept and nutritional management of diabetic kidney disease

Medicine Food Homology (MFH) originates from traditional Chinese medicine, emphasizing the common origins of food and medicine. This concept offers unique advantages in nutritional intervention for diabetic kidney disease (DKD), as its gentle nature renders it particularly suitable for long-term application (266). Recent studies have identified gut microbiota dysbiosis, chronic inflammation, and oxidative stress as core pathological mechanisms underlying DKD progression (209). MFH substances are enriched with bioactive components, including polysaccharides, polyphenols, and flavonoids, which can modulate these pathological processes through multiple molecular targets. Tang et al. (266). systematically reviewed the clinical efficacies and molecular mechanisms of Chinese medicines for diabetic nephropathy, demonstrating their capacity to ameliorate metabolic dysfunction by inhibiting AGEs/RAGE signaling pathways and activating PI3K/AKT/GLUT4 pathways, while concurrently suppressing inflammation and renal fibrosis through modulation of NF-κB and TGF-β/Smad pathways. Hong et al. (221) employed integrated metagenomic and metabolomic analyses to demonstrate that Astragalus polysaccharides can reshape gut microbiota structure, increase the abundance of short-chain fatty acid (SCFA)-producing bacteria, and reduce metabolic endotoxemia by enhancing intestinal barrier function.

Compared with chemically synthesized drugs, MFH substances possess significant safety advantages. The “List of Substances That Are Both Food and Traditional Chinese Medicine” published by China's National Health Commission encompasses over 100 substances with extensive consumption history and favorable safety records. Zhang et al. (267) conducted a systematic review and meta-analysis demonstrating that traditional Chinese medicines used for DKD intervention exhibit good safety profiles with low adverse event rates. Chen et al. (268) systematically elaborated the therapeutic mechanisms of Chinese herbal medicine against DKD, revealing that these medicines exert renoprotective effects through modulating GLP-1 receptor, SGLT2, SIRT1/AMPK, Nrf2, and NLRP3 pathways.

### 7.2 Representative medicine food homology substances

Among MFH substances, Astragalus membranaceus stands as the most widely utilized Qi-tonifying agent with substantial clinical evidence supporting its efficacy in DKD management [Human clinical evidence - meta-analysis level]. Li et al. (269) conducted a meta-analysis encompassing 25 clinical trials with 1,804 patients, demonstrating that Astragalus injection significantly reduced blood urea nitrogen and serum creatinine levels, improved creatinine clearance rate, decreased urinary protein excretion, and elevated serum albumin levels in DKD patients. Lin et al. (270) performed an updated meta-analysis including 32 studies with 2,462 patients, confirming that Astragalus combined with RAAS blockers significantly improved clinical total effective rate (OR 3.63, 95% CI 2.59–5.09) [Evidence quality: Moderate, due to heterogeneity in formulation and dosing].

Mechanistically, Astragalus polysaccharides (APS) attenuate inflammatory responses in diabetic nephropathy by suppressing the TLR4/NF-κB signaling pathway (271) [Animal model evidence], and modulate gut microbiota composition, promoting the proliferation of butyrate-producing bacteria such as Roseburia and Faecalibacterium [Animal model evidence] (221). Notably, while clinical efficacy has been demonstrated, the specific bioactive compounds responsible and their gut microbiota-dependent mechanisms require validation in human intervention studies with microbiome profiling.

Other MFH substances have also demonstrated promising therapeutic potential. Peter et al. (272) conducted a systematic review and meta-analysis demonstrating that bitter melon extract improves glycemic control in type 2 diabetes patients. Crataegus pinnatifida (hawthorn), rich in flavonoid compounds and organic acids, possesses lipid-regulating and antioxidant properties. Studies have revealed that hawthorn polysaccharides can ameliorate lipid metabolism disorders through gut microbiota modulation (273), which may indirectly attenuate lipotoxicity-induced renal injury in DKD.

### 7.3 Application prospects of foods for special medical purposes (FSMP)

Nutritional management of DKD patients necessitates comprehensive consideration of glycemic control, renal function protection, electrolyte balance, and nutritional status maintenance. The KDIGO 2022 Clinical Practice Guideline (274) provides evidence-based nutritional recommendations for patients with diabetes and chronic kidney disease: protein intake of 0.8 g/kg/day for non-dialysis patients (Grade 2C), 1.0–1.2 g/kg/day for hemodialysis and peritoneal dialysis patients, and sodium intake < 2 g/day (Grade 2C). The guideline emphasizes individualized dietary planning, prioritizing vegetables, fruits, whole grains, fiber, legumes, plant-based proteins, unsaturated fats, and nuts.

Plant-based dietary approaches have garnered increasing attention in CKD management. Carrero et al. (275) systematically elaborated the potential benefits of plant-based diets in CKD patients, including reduced uremic toxin production, improved metabolic parameters, and delayed decline in renal function. Rossi et al. (225) conducted the SYNERGY randomized controlled trial in hemodialysis patients, demonstrating that synbiotic intervention significantly reduced serum levels of protein-bound uremic toxins, specifically indoxyl sulfate and p-cresyl sulfate, while concurrently decreasing inflammatory marker levels. These findings underscore the therapeutic potential of integrating MFH substances and functional foods into comprehensive nutritional management strategies for DKD patients.

## 8 Dietary patterns and comprehensive nutritional interventions

### 8.1 Dietary protein management strategies

#### 8.1.1 Individualized adjustment of protein intake

Precise management of protein intake represents a cornerstone of nutritional intervention in DKD. The KDIGO 2024 guideline has updated recommendations for protein intake in patients with CKD: a protein intake of 0.8 g/kg/day is suggested for non-dialysis CKD stages G3–G5 patients, with avoidance of high protein intake (>1.3 g/kg/day). For metabolically stable patients at risk of progression, a very low-protein diet (0.3–0.4 g/kg/day) supplemented with essential amino acids or ketoacid analogs may be considered under close supervision (276). Cheng et al. (277) conducted a systematic review and meta-analysis incorporating six prospective cohort studies with 148,051 participants, demonstrating that higher total protein intake was associated with an 18% reduction in CKD risk (RR = 0.82, 95% CI: 0.71–0.94), plant protein intake with a 23% reduction (RR = 0.77, 95% CI: 0.61–0.97), and animal protein intake with a 14% reduction (RR = 0.86, 95% CI: 0.76–0.97).

From a gut microbiota perspective, Gryp et al. (278) studied 14 healthy controls and 141 CKD patients, demonstrating that while plasma levels of protein-bound uremic toxins increased with CKD progression, fecal concentrations of these toxins and their precursors remained stable. These findings indicate that uremic toxin accumulation primarily results from decreased renal clearance rather than increased intestinal bacterial production. The same research group (279) subsequently isolated and identified 148 bacterial strains from CKD patient feces, of which 92 were uremic toxin precursor-generating bacteria. Carballo-Casla et al. (280) integrated three cohorts comprising 8,543 community-dwelling older adults aged ≥60 years in a prospective follow-up study, finding that higher total protein intake was associated with reduced mortality risk in CKD patients (1.4 vs. 0.8 g/kg/day: HR = 0.73), with both plant and animal protein showing protective effects.

#### 8.1.2 Selection between plant and animal protein

Accumulating evidence supports a plant protein-prioritized dietary strategy. Adeva-Andany et al. (281) conducted a systematic review demonstrating positive associations between animal protein and clinical features of DKD (glomerular hyperfiltration, albuminuria, renal function decline) as well as cardiovascular disease, whereas plant protein exhibited significant protective effects against both conditions. The underlying mechanism is closely related to improved insulin sensitivity associated with plant-based diets. Heo et al. (282), utilizing UK Biobank data from 117,809 participants without baseline CKD over a median 9.9-year follow-up, found that the highest quartile of plant protein intake was associated with an 18% reduction in incident CKD risk (AHR = 0.82, 95% CI: 0.73–0.93), independent of total protein intake.

Alvirdizadeh et al. (283) conducted a prospective study of 1,639 adults from the Tehran Lipid and Glucose Study over a mean 6.1-year follow-up, demonstrating that the highest tertile of plant protein intake was associated with a 71% reduction in CKD risk (OR = 0.29, 95% CI: 0.15–0.55), while total protein and animal protein showed no significant associations, suggesting that protein source is more important than total quantity. The meta-analysis by Cheng et al. (277) further revealed that fish and seafood protein intake was significantly associated with a 16% reduction in CKD risk (RR = 0.84, 95% CI: 0.74–0.94), potentially attributable to the anti-inflammatory effects of omega-3 fatty acids. Based on current evidence, DKD patients are recommended to prioritize plant protein and fish protein while limiting red meat and processed meat consumption.

### 8.2 Dietary fiber and prebiotic supplementation

As the primary energy substrate for gut microbiota, dietary fiber holds a significant position in DKD nutritional intervention. Jia et al. (9) conducted a cross-sectional analysis of 6,032 patients with type 2 diabetes using NHANES 2005–2018 data, finding that the highest tertile of dietary fiber intake (≥10.1 g/1,000 kcal/day) compared with the lowest tertile was associated with a 21% reduction in DKD prevalence (OR = 0.79, 95% CI: 0.68–0.92, *p* = 0.002). Luo et al. (284) analyzed NHANES 2009–2018 data including 4,520 T2DM patients, revealing a nonlinear inverse association between dietary fiber intake and DKD prevalence. Restricted cubic spline analysis identified an inflection point at 13.96 g/day, below which the protective effect of fiber intake was more pronounced. Based on available evidence, a dietary fiber intake of 25–30 g/day is recommended for DKD patients, with soluble fiber comprising 40%−50% of the total.

High-fiber diets exert renal protective effects through multiple mechanisms: increasing the intestinal carbohydrate-to-protein fermentation ratio, thereby reducing production of uremic toxin precursors such as indole and p-cresol; short-chain fatty acids (SCFAs) produced by dietary fiber fermentation, particularly butyrate, enhance intestinal tight junction protein expression, reduce intestinal permeability, and decrease endotoxin translocation into the bloodstream. Additionally, SCFAs modulate immune-inflammatory responses through activation of G protein-coupled receptors.

### 8.3 Mediterranean diet and plant-based dietary patterns

The Mediterranean diet, characterized by abundant plant-based foods, olive oil, moderate fish consumption, and red wine, is recognized as one of the healthiest dietary patterns. Qu et al. (285), utilizing UK Biobank data from 33,441 individuals with hyperglycemia (including 7,969 with T2DM) over a median 12.3-year prospective follow-up, found that the highest compared with the lowest Alternate Mediterranean Diet (AMED) score was associated with a 21% reduction in DKD risk (HR = 0.79, 95% CI: 0.67–0.94). The protective effect was more pronounced in the T2DM subgroup (HR = 0.64, 95% CI: 0.50–0.83). Additionally, each incremental increase in legume consumption was associated with an 8% reduction in DKD risk (HR = 0.92, 95% CI: 0.84–1.01).

Kwon et al. (286) conducted a randomized crossover trial in 28 patients with CKD stages 3–4, demonstrating that a 4-week Mediterranean diet intervention did not result in elevated serum potassium levels (no significant difference pre- and post-intervention) and maintained stable renal function, confirming the safety and feasibility of the Mediterranean diet in CKD patients. Podadera-Herreros et al. (287) performed a secondary analysis of the CORDIOPREV study, including 1,002 patients with coronary heart disease randomized to either a Mediterranean diet or low-fat diet group with 7-year follow-up. In the subgroup with concurrent T2DM and obesity, the Mediterranean diet group exhibited slower eGFR decline compared with the low-fat diet group, suggesting renal protective effects of the Mediterranean diet in high-risk populations.

For Asian populations, adapted protocols integrating core elements of the Mediterranean diet (high plant-based foods, healthy fats, fish) with local traditional ingredients can improve patient adherence. Asian dietary patterns emphasizing whole grains, legumes, vegetables, and fish consumption have demonstrated reduced CKD incidence and progression risk in diabetic patients across multiple prospective cohort studies.

### 8.4 Adjunctive application of probiotics and synbiotics

#### 8.4.1 Evidence hierarchy in probiotic/synbiotic interventions for DKD

The therapeutic potential of probiotics and synbiotics in DKD management is supported by a hierarchical body of evidence spanning mechanistic studies to clinical trials. Understanding the evidence level for each intervention is critical for clinical translation.

#### 8.4.2 Mechanistic evidence (preclinical)

Song et al. (288) demonstrated significantly reduced abundance of *Akkermansia muciniphila* in the intestines of DKD patients and db/db mice [Human observational + animal model evidence]. Exogenous supplementation with this bacterium reduced urinary protein, blood urea nitrogen, and creatinine levels in DKD mice, decreased plasma levels of uremic toxins including TMAO, p-cresyl sulfate, and indoxyl sulfate, and protected intestinal mucosal barrier integrity through upregulation of MUC2, occludin, and ZO-1 protein expression [Animal model evidence only]. While these findings establish biological plausibility, the optimal strain selection, dosing, and duration for human application remain undefined.

#### 8.4.3 Clinical evidence (human studies)

At the clinical evidence level, multiple randomized controlled trials have confirmed the efficacy of probiotic interventions [Human RCT evidence]. Nguyen et al. (289) conducted a meta-analysis of 23 RCTs comprising 931 hemodialysis patients, demonstrating that probiotic/prebiotic/synbiotic interventions significantly reduced plasma p-cresyl sulfate (SMD = −0.38, 95% CI: −0.61 to −0.15), endotoxin (SMD = −0.58), CRP (SMD = −0.61), and IL-6 (SMD = −0.92) [Evidence quality: Moderate - significant heterogeneity in intervention protocols].

Critical evidence gap: most human trials enrolled hemodialysis patients (advanced CKD); evidence for earlier-stage DKD (CKD 1–3) remains limited. Furthermore, strain-specific effects and gut microbiota-dependent response prediction require validation in precision medicine trials.

Table 4 summarizes the studies on natural bioactive compounds and dietary interventions in DKD, along with their corresponding levels of evidence.

**Table 4:** Evidence strength summary for natural bioactive compounds and dietary interventions in DKD.

Intervention	Evidence source	Study design	Sample size/models	Key outcomes	Evidence strength^*^	Clinical translation status
Natural bioactive compounds						
Quercetin	Animal models	Murine hyperuricemic nephropathy model	*N* = 30–50 mice	↓ uremic toxins, ↓ Blautia, ↓ nephrotoxic metabolites	Low	Requires human dose-finding trials
Astragalus polysaccharides (APS)	Human clinical trials + Animal models	Meta-analysis (25 RCTs) + mechanistic studies in mice	*N* = 1,804 patients	↓ BUN, ↓ SCr, ↑ CCr, ↓ proteinuria	Moderate-High	Approved adjunct therapy in China; international trials needed
Berberine	Animal models + small human trials	Diabetic rat models + T2DM patients	*N* = 100 patients (small trials)	Gut microbiota modulation, ↑ Akkermansia	Moderate	Phase II/III DKD trials lacking
Curcumin	Human RCT	T2DM nephropathy patients	*N* = 50–100 patients	↓ proteinuria, ↓ TGF-β, ↓ IL-8	Moderate	Limited by bioavailability; nano-formulations under development
Dietary patterns						
Mediterranean diet	Human cohort studies	UK Biobank prospective cohort	*N* = 33,441 hyperglycemic individuals	21% ↓ DKD risk (HR = 0.79, 95% CI: 0.67–0.94)	Moderate-High	Guideline-recommended; individualization needed for Asian populations
Plant-based protein	Human cohort studies	UK Biobank	*N* = 117,809 participants	18% ↓ incident CKD risk (highest vs. lowest quartile)	Moderate	Observational; RCTs for DKD-specific outcomes needed
Dietary fiber (25–30 g/day)	Human cross-sectional + RCTs	NHANES analysis + intervention trials	*N* = 6,032 T2DM patients	21% ↓ DKD prevalence; ↑ SCFAs	Moderate	Guideline-recommended; optimal fiber type requires clarification
Probiotics/prebiotics/synbiotics						
*Akkermansia muciniphila* supplementation	Animal models	db/db diabetic mice	*N* = 20–30 mice	↓ proteinuria, ↓ TMAO, ↓ pCS, ↑ tight junction proteins	Low	No human DKD trials; safety/efficacy in humans unknown
Multi-strain probiotics	Human RCTs (hemodialysis patients)	Meta-analysis (23 RCTs)	*N* = 931 hemodialysis patients	↓ pCS, ↓ CRP, ↓ IL-6, ↑ antioxidant capacity	Moderate	Limited to dialysis populations; early-stage DKD data lacking
Resistant starch (RS2)	Human RCT	CKD stage 3a-4 patients	*N* = 50–100 patients	↓ uremic toxins, gut microbiota modulation	Moderate	Small sample sizes; long-term safety/efficacy unclear
Functional foods						
Synbiotics (SYNERGY trial)	Human RCT	Hemodialysis patients	*N* = 42 patients	↓ IS, ↓ pCS, ↓ IL-6, ↓ TNF-α	Moderate	Narrow population; DKD-specific formulations needed
Inulin supplementation	Human observational + small RCTs	CKD patients	*N* = 50–100 patients	↑ Bifidobacterium, ↓ urease-producing bacteria	Low-Moderate	Dose-response relationship undefined; GI tolerability issues

Evidence strength grading: (1) High: multiple large RCTs with consistent results; low heterogeneity; meta-analysis support; (2) Moderate: some RCTs or large cohort studies; moderate heterogeneity; mechanistic support; and (3) Low: primarily animal models or small uncontrolled human studies; high heterogeneity. Key evidence gaps requiring future research: (1) Strain-specific probiotic effects in early-stage DKD (CKD 1–3); (2) Long-term safety and efficacy of high-dose polyphenols in humans; (3) Gut microbiota-stratified precision nutrition trials; and (4) Comparative effectiveness of natural compounds vs. SGLT2 inhibitors/GLP-1 Ras. ^*^This symbol denotes the most critical information presented in this table.

#### 8.4.4 Translation of preclinical evidence to clinical practice: critical methodological gaps

A systematic evaluation of the evidence base presented in this review reveals significant translational barriers between preclinical mechanistic studies and clinical efficacy validation.

Of the natural bioactive compounds discussed, fewer than 30% have been evaluated in adequately powered randomized controlled trials in human DKD populations. The majority of renoprotective claims derive from rodent models (primarily db/db mice, streptozotocin-induced diabetes, or high-fat diet models), which exhibit fundamental differences from human DKD in gut microbiota composition, metabolic kinetics, and disease progression timelines.

**Interspecies variability in gut microbiota responses:** rodent gut microbiota is dominated by Firmicutes and Bacteroidetes at ratios differing markedly from human populations, and lacks key human-enriched genera such as *Prevotella* and *Blautia*. Consequently, polyphenol biotransformation, SCFA production ratios, and uremic toxin generation pathways observed in mice may not accurately predict human responses. For example, while quercetin supplementation selectively enriched *Akkermansia muciniphila* and reduced uremic toxins in murine models (211), human bioavailability is < 10% due to extensive first-pass metabolism, and dose-escalation trials with microbiome profiling are lacking.

**Dose extrapolation challenges:** preclinical studies frequently employ doses that, when scaled allometrically to human equivalents, exceed feasible oral intake by 5–10 fold. Astragalus polysaccharides administered at 200–400 mg/kg/day in mice (221) would translate to ~14–28 g/day in a 70-kg human—a dose rarely achieved even in concentrated extract formulations. This dose-response discordance complicates clinical translation and necessitates pharmacokinetic bridging studies.

**Recommendations for evidence hierarchy in future research:** moving forward, we propose a structured translational pipeline:

**Phase I**: mechanistic validation in human-relevant models (e.g., human gut microbiota-colonized mice, organoid co-cultures)

**Phase II**: pharmacokinetic/pharmacodynamic studies in healthy volunteers with microbiome profiling

**Phase III**: proof-of-concept RCTs in early-stage DKD (CKD 1–3) with prespecified microbiome-stratification

**Phase IV**: comparative effectiveness trials vs. standard-of-care therapies (SGLT2i, ACEi/ARBs)

**Current evidence strength assessment:** based on GRADE criteria applied to the interventions reviewed:

- **High-quality evidence**: mediterranean diet, plant-based protein (large cohorts, consistent epidemiological associations)
- **Moderate-quality evidence**: Astragalus (multiple RCTs, but heterogeneity in formulation), probiotics in dialysis (RCTs with moderate risk of bias)
- **Low-quality evidence**: most single-compound polyphenols, prebiotics, individual bacterial strains (animal models predominate, human data sparse or underpowered).

This evidence stratification should guide clinical recommendations, prioritizing interventions with robust human data while appropriately framing mechanistically promising but clinically unproven compounds as requiring further investigation.

Second, natural active compounds exert renal protective effects through modulation of amino acid metabolism [Evidence: Primarily animal models with limited human validation]. Natural active compounds including polysaccharides, polyphenols, and flavonoids can modulate gut microbiota structure and influence metabolic pathways such as tryptophan-indole and tyrosine-p-cresol [Animal model evidence]. Kikuchi et al. (112) confirmed that gut microbiota-derived phenyl sulfate is an important contributor to albuminuria in diabetic kidney disease [Human mechanistic study], suggesting that uremic toxin production and absorption can be reduced through modulation of gut microbiota metabolism. This microbiota-metabolite-target organ regulatory paradigm provides new perspectives for understanding the mechanisms of action of natural active compounds. However, clinical translation requires dose-finding studies, pharmacokinetic validation, and adequately powered randomized controlled trials in human DKD populations to establish efficacy and safety.

#### 8.4.5 Evidence strength and clinical translation readiness

Critical appraisal of the evidence base reveals substantial heterogeneity in research maturity across interventions. Dietary pattern modifications (Mediterranean diet, plant protein prioritization) are supported by large-scale epidemiological studies and align with existing clinical practice guidelines, representing immediately implementable strategies. In contrast, most single-compound natural products remain at the preclinical validation stage, with mechanistic evidence derived predominantly from rodent models. The field urgently requires:

1. Translational pharmacology studies bridging animal model doses to safe and effective human equivalents
2. Gut microbiota-stratified clinical trials identifying responder phenotypes for precision nutrition
3. Comparative effectiveness research evaluating natural compounds as adjuncts to SGLT2 inhibitors and RAAS blockade
4. Long-term safety surveillance for high-dose polyphenol and probiotic supplementation in CKD populations.

Until robust human data emerge, clinicians should prioritize evidence-based dietary modifications (fiber, plant protein, Mediterranean patterns) while cautiously integrating mechanistically promising but clinically unproven natural compounds only within research protocols or shared decision-making frameworks that explicitly acknowledge current evidence limitations.

## 9 Challenges, perspectives and clinical translation

### 9.1 Major challenges in current research

Despite numerous observational studies revealing associations between gut microbiota and diabetic kidney disease (DKD), establishing causal relationships remains methodologically challenging. Mendelian randomization (MR) studies provide new tools for this purpose. Zhang et al. (290) employed bidirectional MR analysis to evaluate the causal relationship between gut microbiota and DKD, utilizing genome-wide association study data from the MiBioGen consortium as genetic instrumental variables for gut microbiota, and found causal associations between specific bacterial genera and DKD risk. Fang et al. (291) further confirmed causal relationships between specific genera such as *Eubacterium* and the development and progression of diabetic nephropathy. However, MR studies are limited by insufficient power of genetic instrumental variables for microbiota and potential pleiotropic bias, requiring cautious interpretation of conclusions.

Translatability of animal models represents another critical issue. Significant differences exist between rodent and human gut microbiota composition, often resulting in attenuated effects when translating animal experimental results to clinical applications. Insufficient standardization of intervention studies exacerbates difficulties in evidence synthesis. Liu et al. (292) conducted a systematic review including 21 randomized controlled trials and found substantial heterogeneity in existing probiotic/synbiotic intervention studies regarding strain selection, dosage, and duration. Although overall results showed that probiotics/synbiotics significantly reduced blood urea nitrogen and C-reactive protein levels, individual effect variations were substantial.

Large inter-individual variations in gut microbiota represent a key factor affecting intervention efficacy. Berry et al. (293) recruited 1,002 twins and healthy adults in the PREDICT 1 study and found significant inter-individual variability in postprandial glucose, triglyceride, and insulin responses to identical meals (coefficients of variation of 68, 103, and 59%, respectively), with gut microbiome composition explaining much of this variation (7.1% of variance). Such heterogeneity similarly exists in DKD populations. The meta-analysis by Zheng et al. (294) demonstrated that probiotic/prebiotic/synbiotic interventions improved inflammation and oxidative stress markers in CKD patients, but with significant inter-study effect variations, suggesting that baseline microbiota characteristics may influence intervention responses.

Low bioavailability of natural active compounds represents an important bottleneck limiting clinical application. Most polyphenols, polysaccharides, and flavonoids have oral bioavailability below 10%. Jin et al. (295) noted that polyphenolic compounds exert protective effects in diabetic nephropathy by modulating oxidative stress and inflammatory responses, but are extensively metabolized by gut microbiota in the intestine, with secondary metabolites potentially having completely different biological activities from parent compounds. Therefore, evaluating the efficacy of natural active compounds requires consideration of their metabolite profiles. Insufficient renal targeting represents another challenge; modern formulation technologies such as nanocarriers can improve renal targeting but also increase production costs and regulatory complexity.

Long-term intervention adherence represents a practical barrier to clinical translation. Rysz et al. (296) noted that CKD patients face complex dietary restrictions and multiple drug treatments, with low long-term adherence rates to complex dietary regimens, primarily hindered by poor taste acceptability and difficulties in lifestyle modification. Developing functional food formulations with good palatability and convenient administration represents an important strategy for improving adherence.

### 9.2 Future directions in precision nutrition

The core of precision nutrition is developing personalized intervention strategies based on individual characteristics. Ratiner et al. (297) systematically elaborated on the application prospects of gut microbiota in personalized medicine, noting that due to significant inter-individual variability and person-specific signatures of the microbiome, microbiota data can serve as important biomarkers for early disease detection, prognostic assessment, and personalized treatment. Enterotype classification provides a foundational framework for individualized intervention, with systematic differences in responses to dietary interventions among different enterotypes. Machine learning algorithms provide powerful tools for modeling microbiota-diet-disease relationships, with similar strategies applicable to predicting DKD nutritional intervention effects.

Multi-omics integration analysis provides systematic guidance for precision nutrition formulation design. Simon et al. (298) noted that comprehensive analysis of individual nutritional requirements and metabolic characteristics can be achieved by integrating genomic, transcriptomic, metabolomic, and microbiome data. Metabolomic biomarkers can be used to monitor intervention effects and guide regimen adjustments. The meta-analysis by Chen et al. (299) demonstrated that probiotic/prebiotic/synbiotic supplementation reduced C-reactive protein, interleukin-6, and indoxyl sulfate levels in dialysis patients, suggesting that monitoring systems for uremic toxins and inflammatory markers can provide real-time assessment of gut microbiota functional status to guide dynamic adjustment of nutritional regimens.

The development of digital health technologies brings revolutionary opportunities for DKD nutritional management. The popularization of wearable devices and continuous glucose monitoring technology makes real-time metabolic monitoring possible. Zeevi et al. (300) developed a personalized glycemic response prediction system integrating glucose monitoring, dietary records, anthropometric indices, physical activity, and microbiome data, which accurately predicts individual glycemic responses to specific foods through machine learning algorithms (*r* = 0.77). A randomized controlled trial validated that personalized dietary interventions based on this algorithm significantly reduced postprandial glycemic responses. AI-driven dietary recommendation systems can automatically generate personalized meal plans based on patients' clinical indicators and microbiota characteristics, with preliminary applications showing that AI-assisted nutritional management significantly improves patient adherence.

#### 9.2.1 Cost-effectiveness-oriented multi-omics implementation

##### 9.2.1.1 Economic constraints and technological assessment

Single comprehensive multi-omics profiling—encompassing metagenomics, metabolomics, transcriptomics, and proteomics—incurs costs exceeding $2,000–5,000, rendering its application impractical for chronic disease management requiring sustained longitudinal surveillance. Despite demonstrated efficacy of synbiotic interventions in dialysis populations, economic evaluation studies remain conspicuously absent **[**299**]**, underscoring the imperative for establishing pragmatic, financially sustainable screening frameworks.

**Comparative economic analysis of microbiome profiling methodologies**: 16S rRNA amplicon sequencing ($50–150 per specimen) delivers genus-level taxonomic discrimination adequate for detecting depleted short-chain fatty acid (SCFA)-producing genera *Faecalibacterium* and *Roseburia*, alongside expanded uremic toxin-generating *Escherichia-Shigella* populations (98, 190). Diagnostic sensitivity for diabetic kidney disease (DKD) discrimination (AUC 0.78–0.85) approaches 80% of shotgun metagenomic sequencing ($300–800 per specimen) performance at one-fifth the expenditure (290, 291). Shotgun approaches enable species-level resolution and functional gene cataloging, revealing enhanced tryptophanase (*tnaA*) expression in indole-producing bacterial strains (117), warranting reservation for treatment-refractory cases or mechanistic research cohorts.

**Metabolomics platform selection**: untargeted metabolomic profiling ($400–1,200 per specimen) comprehensively surveys thousands of metabolites but generates complex datasets necessitating specialized bioinformatics infrastructure. Targeted metabolomic panels quantifying predefined uremic toxins [indoxyl sulfate (IS), p-cresyl sulfate (pCS), trimethylamine N-oxide (TMAO), phenylacetylglutamine] and SCFAs cost $150–300 per specimen with superior analytical precision (coefficient of variation < 15% vs. 20%−35%) (136, 171). Meta-analytic evidence identifies lactate, hippuric acid, urea, and glutamine as diagnostically optimal early-stage DKD biomarkers (153), suggesting that focused analytical panels capturing 15–20 critical analytes satisfy clinical decision-making requirements.

##### 9.2.1.2 Three-tiered minimum viable dataset architecture

**Tier 1 (foundation stratum, $0–50 per patient)**: routinely collected clinical parameters encompass estimated glomerular filtration rate (eGFR), urinary albumin-to-creatinine ratio (UACR), glycated hemoglobin (HbA1c), fasting plasma glucose, lipid profile, and serum homocysteine. These conventional markers stratify DKD risk and monitor disease trajectory without incremental financial burden. Plasma homocysteine integrated with clinical metrics demonstrates moderate predictive accuracy for chronic kidney disease (CKD) risk (odds ratio 1.17 per standard deviation increment) (147).

**Tier 2 (precision stratum, $200–400 per patient)**: designated for progressive DKD presentations (eGFR decline >3 mL/min/1.73 m^2^/year or persistent macroalbuminuria). Cost-optimized multi-omics screening incorporates: (1) fecal 16S rRNA microbiome profiling for enterotype classification and dysbiosis severity quantification; (2) targeted fecal SCFA quantification (acetate, propionate, butyrate) via gas chromatography-mass spectrometry (GC-MS); and (3) serum-targeted metabolomics panel measuring protein-bound uremic toxins (IS, pCS, TMAO) and branched-chain amino acids (BCAAs). This stratum furnishes actionable data for personalized dietary prescription at approximately one-tenth comprehensive profiling expenditure. Fecal SCFAs exhibit robust inverse correlations with UACR (*r* = −0.38, *p* < 0.01) (191), substantiating clinical utility. Machine learning architectures integrating 16S rRNA data with serum BCAA measurements (aggregate cost ~$250) achieve predictive accuracy (AUC 0.832) comparable to comprehensive multi-omics profiling (>$2,000) for DKD vs. control discrimination (152). Given functional redundancy whereby multiple bacterial taxa generate identical uremic toxin precursors, serum metabolite quantification (IS, pCS) suffices for therapeutic surveillance, reserving microbiome profiling for baseline characterization (278).

**Tier 3 (research stratum, $1,000–3,000 per patient)**: reserved exclusively for clinical trials, refractory presentations, or academic investigations. Encompasses shotgun metagenomic sequencing with functional gene annotation, untargeted metabolomics, bile acid profiling, and transcriptomics. Ultra-high-performance liquid chromatography-tandem mass spectrometry (UHPLC-MS/MS) characterizes stepwise bile acid perturbations across DKD stages (199), demonstrating research value but constrained routine applicability given costs exceeding $800 per analysis.

##### 9.2.1.3 Artificial intelligence-mediated dataset minimization

Machine learning algorithms retrospectively extract minimal feature sets retaining >90% predictive capacity from comprehensive multi-omics repositories. Dimensionality reduction methodologies compress metabolomic biomarker panels from 200–500 features to 15–30 critical analytes without significant accuracy attrition (298). Personalized glycemic response prediction systems incorporating merely seven microbiome features (selected from >1,000 bacterial taxa) combined with dietary and anthropometric variables achieve robust correlation (*r* = 0.77) with continuous glucose monitoring (300).

Continuous glucose monitoring coupled with microbiome-informed dietary modifications enhances adherence and glycemic regulation (coefficient of variation reduction from 68 to 42%) (293). Microbiome stability in healthy populations obviates frequent re-profiling; dysbiosis correction verification at 3–6 month intervals satisfies treatment optimization requirements (297).

### 9.3 Clinical translation and application prospects

Combined application of natural active compounds with modern drugs represents an important strategy for enhancing efficacy and reducing side effects. SGLT2 inhibitors, as first-line therapy for DKD, have attracted attention for their combined use with natural active compounds. Neuen et al. (301) demonstrated that combination treatment with SGLT2 inhibitors and other cardio-renal protective agents significantly extends cardiovascular event-free, kidney event-free, and mortality-free survival in patients with type 2 diabetes and albuminuria. Polyphenolic compounds such as resveratrol and quercetin exert renal protective effects through activation of multiple pathways including Nrf2, AMPK, and SIRT1 (302). Zhao et al. (183) systematically reviewed the mechanisms of gut microbiota and their metabolites in diabetic nephropathy, providing a theoretical basis for targeted intervention with natural active compounds.

The positioning of functional foods in DKD management should be as beneficial supplements to drug therapy rather than substitutes. Early DKD (microalbuminuria stage) represents the optimal window for functional food intervention, during which medicinal-food homology functional foods can significantly delay disease progression. Cooper et al. (303) conducted a Cochrane systematic review including 45 randomized controlled trials with 2,266 participants, evaluating the effects of synbiotics, prebiotics, and probiotics in CKD management. Although certainty of existing evidence was low, adverse events were uncommon and mild, supporting further high-quality clinical trials.

Current DKD nutritional management guidelines primarily focus on macronutrients and electrolytes, with relatively insufficient guidance on gut microbiota modulation strategies. Based on the latest evidence-based findings, it is recommended to incorporate the following into guidelines: recommended intake of dietary fiber and prebiotics (25–30 g/day); indications and strain recommendations for adjunctive probiotic application; and recommendations for promoting plant-based dietary patterns. These updates will provide more comprehensive guidance for clinical practice.

With SGLT2 inhibitors established as the cornerstone pharmacotherapy for DKD, careful evaluation of potential nutrient-drug interactions accompanying high-dose polyphenolic supplementation becomes imperative. Although direct clinical investigations specifically addressing SGLT2 inhibitor-polyphenol interactions remain scarce, accumulating theoretical frameworks and preliminary experimental observations warrant clinical vigilance.

Quercetin and structurally related flavonoids demonstrate inhibitory activity against cytochrome P450 isoenzymes (notably CYP3A4) alongside P-glycoprotein-mediated efflux mechanisms (304, 305). Hepatic biotransformation of SGLT2 inhibitors proceeds predominantly via UDP-glucuronosyltransferase pathways, with selected agents exhibiting partial CYP3A4 involvement (306). Quercetin administration exceeding 500 mg daily may theoretically modulate systemic SGLT2 inhibitor exposure through metabolic pathway interference, though clinical ramifications remain undefined. Polyphenolic constituents such as epigallocatechin gallate exhibit competitive antagonism toward intestinal SGLT1 transporters (307).

Both SGLT2 inhibitors and polyphenolic agents manifest convergent antioxidant and anti-inflammatory properties. SGLT2 inhibitors attenuate oxidative stress through multifaceted mechanisms encompassing glucotoxicity reduction and mitochondrial functional enhancement (308). Quercetin activates AMP-activated protein kinase signaling cascades that partially intersect with SGLT2 inhibitor metabolic pathways (309). Such mechanistic convergence theoretically potentiates therapeutic efficacy while simultaneously elevating hypoglycemia susceptibility among patients receiving concurrent antidiabetic regimens.

Notably, SGLT2 inhibitors elicit mild osmotic diuresis and perturb electrolyte homeostasis, particularly affecting magnesium and phosphate metabolism (310). Certain polyphenolic compounds possess intrinsic diuretic properties that may exacerbate volume depletion or electrolyte derangements when combined with SGLT2 inhibitors. Furthermore, SGLT2 inhibition promotes fatty substrate utilization and ketogenesis (311). In susceptible individuals, this metabolic shift potentially amplifies the theoretical risk of euglycemic diabetic ketoacidosis. Consequently, DKD patients receiving SGLT2 inhibitor therapy should engage healthcare providers before initiating high-dose polyphenolic supplementation (quercetin >500 mg/day or equivalent flavonoid doses). Recommended surveillance protocols encompass: (1) intensified glycemic monitoring throughout the initial 2–4 weeks of combined intervention; (2) serial assessment of renal function indices and electrolyte panels (emphasizing potassium and magnesium); and (3) patient education regarding ketoacidosis symptomatology in high-risk cohorts. Antidiabetic medication titration may prove necessary contingent upon individual glycemic responses.

Future investigational priorities should encompass: (1) rigorously designed pharmacokinetic evaluations examining standardized polyphenolic extract effects on SGLT2 inhibitor plasma kinetics; (2) randomized controlled trials assessing safety and efficacy profiles of combined SGLT2 inhibitor and high-dose polyphenol interventions in DKD populations; and (3) identification of patient subgroups most likely to derive benefit or experience adverse outcomes from combination strategies. Pending robust clinical evidence generation, a prudent individualized approach incorporating appropriate monitoring remains warranted.

## 10 Conclusions

This review systematically elaborates on the latest research advances in gut microbiota, natural active compounds, and nutritional interventions for diabetic kidney disease. The main conclusions are as follows:

First, the gut microbiota-gut-kidney axis serves as a critical nexus connecting dietary nutrition and DKD. DKD patients exhibit significant gut microbiota dysbiosis, characterized by reduced short-chain fatty acid-producing bacteria, increased proteolytic bacteria, and impaired intestinal barrier function. Graboski et al. (312) systematically elaborated on the production mechanisms of gut-derived protein-bound uremic toxins. This dysbiosis leads to increased production of uremic toxins such as indoxyl sulfate and p-cresyl sulfate. Chen et al. (313) further revealed the structural and functional characteristics of gut microbiota in DKD patients and their associations with systemic inflammation and renal injury progression. Nutritional intervention strategies targeting gut microbiota have opened new avenues for DKD management.

Second, natural active compounds exert renal protective effects through modulation of amino acid metabolism. Natural active compounds including polysaccharides, polyphenols, and flavonoids can modulate gut microbiota structure and influence metabolic pathways such as tryptophan-indole and tyrosine-p-cresol. Kikuchi et al. (112) confirmed that gut microbiota-derived phenyl sulfate is an important contributor to albuminuria in diabetic kidney disease, suggesting that uremic toxin production and absorption can be reduced through modulation of gut microbiota metabolism. This microbiota-metabolite-target organ regulatory paradigm provides new perspectives for understanding the mechanisms of action of natural active compounds.

Third, the concept of medicinal-food homology and functional foods provides new strategies for DKD nutritional intervention. The review by Aryal et al. (302) systematically summarized the therapeutic potential of dietary phenolic compounds in the management of diabetes and its complications. Medicinal-food homology substances possess both nutritional value and therapeutic efficacy, with good safety profiles suitable for long-term application. Functional foods developed using modern formulation technologies can improve the bioavailability and targeting of active compounds, serving as bridges connecting traditional dietary wisdom with modern precision medicine.

Fourth, precision nutrition and multidisciplinary collaboration represent future directions. Li et al. (20) confirmed that dietary fiber protects against diabetic nephropathy through short-chain fatty acid-mediated activation of G protein-coupled receptors GPR43 and GPR109A, providing a mechanistic basis for individualized intervention based on gut microbiota characteristics. Multi-omics-guided precision formulation design and the application of intelligent nutritional management systems will drive the transformation of DKD nutritional intervention from empirical medicine to precision medicine. Multidisciplinary collaboration among clinical nutritionists, nephrologists, and food scientists provides the organizational foundation for achieving this transformation.

In summary, gut microbiota modulation and natural active compound intervention hold significant value and broad prospects in DKD nutritional management. Future research should focus on addressing key scientific questions including confirmation of causal relationships, management of individual variability, and improvement of bioavailability, accelerating clinical translation to provide safer, more effective, and personalized nutritional intervention strategies for DKD patients.
